# Fluorescent molecular rotors as versatile in situ sensors for protein quantitation

**DOI:** 10.1038/s41598-023-46571-5

**Published:** 2023-11-22

**Authors:** Kevin Daus, Sorachat Tharamak, Wanchai Pluempanupat, Peter A. Galie, Maria A. Theodoraki, Emmanuel A. Theodorakis, Mary L. Alpaugh

**Affiliations:** 1 Department of Biological and Biomedical Sciences, Rowan University, 201 Mullica Hill Rd, Glassboro, NJ 08028 USA; 2 Department of Chemistry and Biochemistry, University of California, San Diego, 9500 Gilman Drive, La Jolla, CA 92093-0358 USA; 3 Department of Chemistry and Center of Excellence for Innovation in Chemistry, Special Research Unit for Advanced Magnetic Resonance, Faculty of Science, Kasetsart University, Bangkok, 10900 Thailand; 4 Department of Biomedical Engineering, Rowan University, Glassboro, NJ 08028 USA; 5 Department of Biology, Arcadia University, 450 S. Easton Rd, Glenside, PA 19038 USA

**Keywords:** Biological techniques, Chemical biology

## Abstract

Accurate protein quantitation is essential for many cellular mechanistic studies. Existing technology relies on extrinsic sample evaluation that requires significant volumes of sample as well as addition of assay-specific reagents and importantly, is a terminal analysis. This study exploits the unique chemical features of a fluorescent molecular rotor that fluctuates between twisted-to-untwisted states, with a subsequent intensity increase in fluorescence depending on environmental conditions (e.g., viscosity). Here we report the development of a rapid, sensitive in situ protein quantitation method using **ARCAM-1**, a representative fluorescent molecular rotor that can be employed in both non-terminal and terminal assays.

## Introduction

The development of accurate methods to measure protein content is essential for the study of many cellular processes^1–4^ and applicable to various areas of basic and translational science^5–9^. Selecting a suitable assay depends greatly on the level of sensitivity and specificity that is required for the designed study^10^. Existing technologies can be grouped into four different categories that include: (a) direct UV absorption measurements of a protein sample; (b) visualizing redox reactions of the protein in the presence of a chromogenic molecule; (c) using protein-binding assays with colorimetric or fluorescent dyes; and (d) mass spectrometry-based methods^11–13^. Each of these technologies has strengths and weaknesses that require extensive evaluation and fine-tuning prior to use. For instance, the UV absorption technique measures the amount of aromatic amino acids in a protein sample. Albeit simple and straight-forward, this assay suffers from interference by nucleic acids and buffers that absorb in the nearby region. As such, this assay is most advantageous with well-characterized and homogeneous protein preparations. However, most protein quantitation studies are performed on heterogeneous protein solutions and therefore require more versatile assays. The second category involves reduction of Cu(II) to Cu(I) by oxidizable amino acids of the protein. The resulting Cu(I) species is then visualized using inorganic acids (Folin-Lowry assay)^14,15^ or bicinchoninic acid (BCA assay)^16^. Notwithstanding their sensitivity, these assays have limited compatibility with commonly used reagents/buffers and are also terminal since they irreversibly damage the protein sample. The third category relies on colorimetric and fluorescent techniques. In general, the choice of such assays depends upon volume of experimental sample (i.e., expendability), sensitivity and experimental solution compatibility with the dyes. For example, the well-known Bradford assay is based on Coomassie Blue that upon binding to a protein changes its color from red (acidified and unbound state) to blue (anionic and bound state)^17^. Albeit rapid and sensitive, this assay is not compatible with substances used in protein extraction buffers, such as detergents (in high concentrations), and requires the development of a titration curve with appropriate control samples since its response varies widely as a function of the protein structure^2,3,15^. One general limitation of all these assays is the inability to be performed in situ and over long durations of time without compromising cell viability of the biological system or protein activity of the sample. This limitation presents a challenge that can be overcome by exploiting the intrinsic fluorescent properties of molecular rotors^18^.

**Figure 1:**
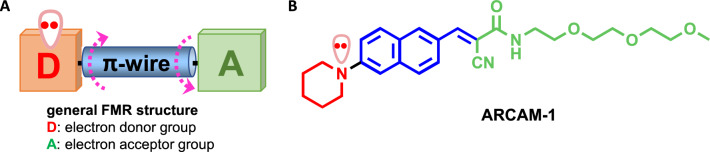
Fluorescent molecular rotor mechanism and structure. (**A**) General structure of a fluorescent molecular rotor (FMR) where an electron donor group [D] is connected with an electron acceptor group [A] via a π-wire. The dashed arrows indicate possible rotation sites that facilitate non-fluorescent mechanical de-excitation of the probe in environments of low protein content. (**B**) Chemical structure of **ARCAM-1,** a representative FMR.

Fluorescent molecular rotors (FMRs) are small molecules that display an environment-sensitive fluorescence emission profile^19–21^. Their chemical structure features an electron donor (D) and an electron acceptor (A) group that are connected via a motif of alternating double and single bonds (π-wire) (Fig. 1A). Alignment of all π orbitals brings the molecule to a planar ground state (i.e., conjugation) that allows movement of electron density between D and A. During photoexcitation, the FMR absorbs energy by jumping to an excited state in which the electron density (e.g., a lone electron pair located on D) has relocated from D to A. De-excitation can occur via either radiative (i.e., fluorescence emission) or non-radiative processes (i.e., intramolecular rotation across the σ-bonds that connect D with A) the ratio of which depends on the surrounding microenvironment. When the intramolecular rotation becomes hindered (e.g., in a viscous or rigid environment), the molecule cannot de-excite by mechanical processes and thus the intensity of its fluorescence increases^22^. On the other hand, FMR de-excitation in a fluid environment (e.g., in a low viscosity or high free volume) leads to both fluorescence emission and mechanical relaxation processes, the ratio of which is related to the fluidity of the medium. Along these lines, the intensity of the fluorescence emission of FMRs can be correlated to the viscosity and/or molecular crowding of their microenvironment. Due to these properties, FMRs have been used to study environment changes in various organized assemblies including liposomes, cells, polymers and protein aggregates^23,24^. Importantly, FMRs can be tailored to have a non-specific affinity for hydrophobic pockets or regions of proteins^25^. Positioning of the FMR within the hydrophobic pocket hinders molecular rotation, subsequently producing an increase in fluorescence emission that is proportional to the molecular crowding of the solution.

We hypothesized that, by virtue of their environment-sensing ability, FMRs can be used as tools for protein quantitation in both extrinsic and in situ analyses of biological samples. We selected **ARCAM-1** (Fig. 1B) as a representative FMR due to its synthetic accessibility, advantageous photophysical properties and sufficient solubility in aqueous environments^26^. In this study we: (i) performed spectral characterization of **ARCAM-1;** (ii) identified a linear correlation between protein concentration (of both homogeneous and heterogeneous samples) and fluorescence intensity increase of **ARCAM-1**; and (iii) generated a standard curve to determine the protein concentration of an unknown test sample. Significantly, we found that **ARCAM-1** performed comparably to the colorimetric bicinchoninic acid (BCA) assay in accurately identifying the protein concentration of an unknown sample. We were also able to track an interstitial fluid flow front through a hydrogel filled perfusable microfluidic device and demonstrated the cellular compatibility of **ARCAM-1** over a 24-h period.

## Results and discussion

### Mechanism of FMR-based environment sensing

FMRs are structurally distinguished by the presence of an electron donor (D) group that is connected via a network of alternating double and single bonds (π-wire) with an electron acceptor (A) group (Fig. 1A). This design is exemplified in the structure of **ARCAM-1** where the piperidine nitrogen (i.e., electron donor) is connected with the cyanoacrylate motif (i.e., electron acceptor) via a naphthalene unit (i.e., π-wire) (Fig. 1B). The π orbital of the piperidine nitrogen that contains the lone electron pair is aligned with all π orbitals of both the π-wire and the acceptor group. During photoexcitation, the lone electron pair of the piperidine nitrogen relocates from D to A and subsequently during relaxation it returns back to D. Various environmental factors affect the energies of the ground and excited states shifting the fluorescence emission wavelength. Relaxation from the excited state can occur via two competing pathways: fluorescence emission and non-fluorescent mechanical de-excitation (e.g., intramolecular rotation of the D and A groups around the π-wire). Constraints of molecular rotation lead to a significant increase in fluorescence emission, which intensifies proportionally to the viscosity or the molecular crowding of the solution (i.e., environment).

### FMR spectral analysis in various aqueous solutions

**Figure 2:**
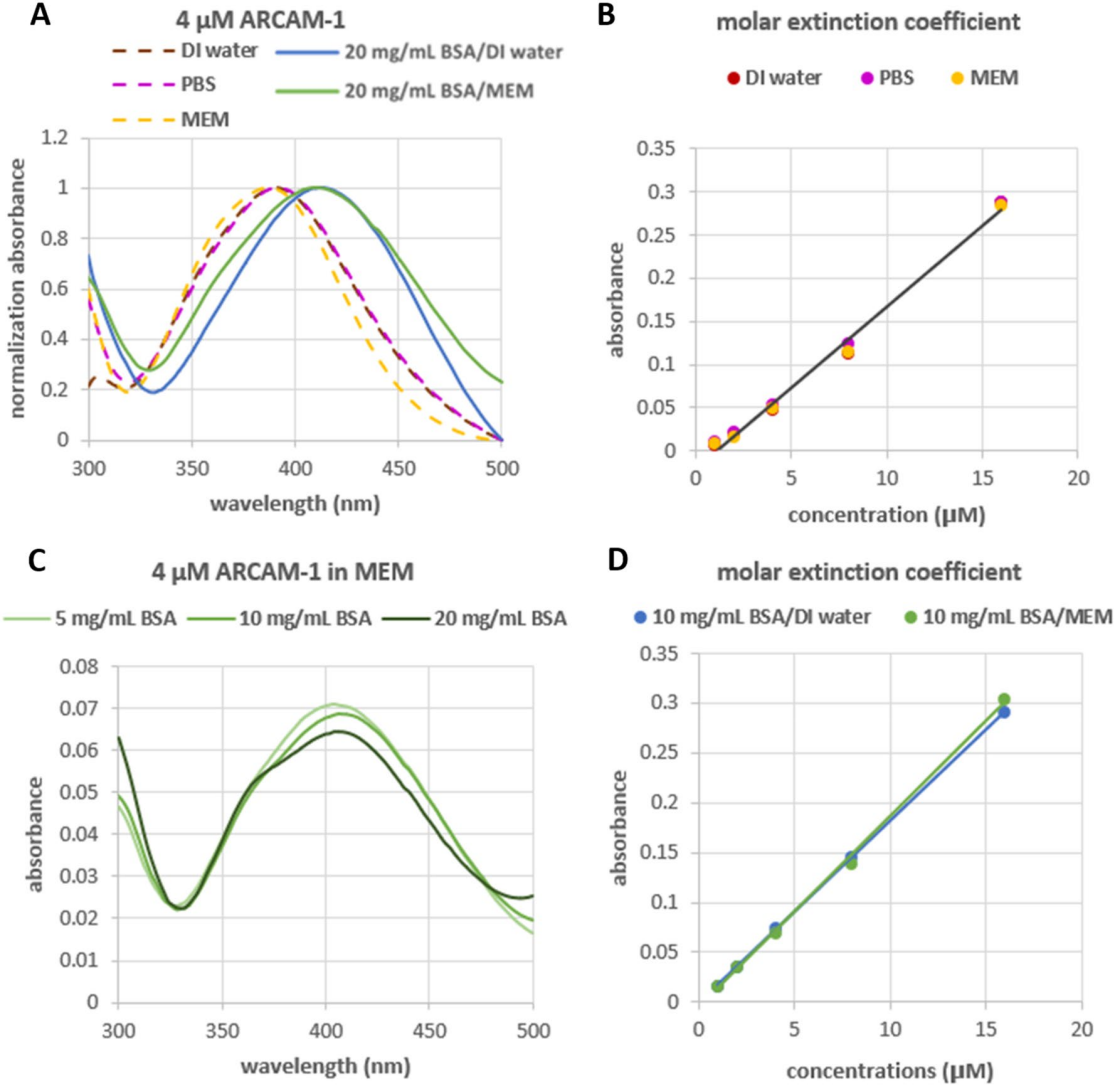
Absorbance spectra of **ARCAM-1** in various aqueous solutions. Absorbance in the absence or presence of BSA is represented with dashed and solid lines, respectively. (**A**) Normalized absorbance spectra of **ARCAM-1** (4 µM). (**B**) Molar extinction coefficient of **ARCAM-1**. (**C**) Absorbance spectra of **ARCAM-1** at 4 µM in presence of different concentrations BSA (5, 10, and 20 mg/mL) in MEM as the solvent. (**D**) Molar extinction coefficient of **ARCAM-1** when bound with BSA (10 mg/mL) in DI water and MEM solutions.

The absorbance of **ARCAM-1** was characterized in various aqueous solutions, including deionized water (DI), phosphate buffered saline (PBS) and minimal essential medium (MEM) at different final FMR concentrations ranging from 1 to 16 µM. The maximum absorption peak of **ARCAM-1** was observed at 372–390 nm (Fig. 2A and Table SI1). The relationship between absorption and **ARCAM-1** concentrations followed linear plots, in accordance with the Beer-Lambert law, with a consistent molar extinction coefficient value of 1.9 × 10^4^ M^-1^ cm^-1^ (Fig. 2B and Table SI1). To investigate whether FMR-protein association would alter the spectral parameters, absorbance measurements were also conducted in the presence of bovine serum albumin (BSA; 20 mg/mL), a representative protein, using DI water and MEM as solvents. The results showed that the maximum absorbance peaks in the presence of BSA exhibited a bathochromic shift to 409–410 nm in both solvents (Fig. 2A). This trend is due to the negative solvatochromism, a property known for this type of dyes that undergo a red shift in their absorption spectra upon decrease of the polarity of their environment (e.g., binding to hydrophobic protein sites)^27,28^.

Moreover, the absorbance spectra at different concentrations of BSA (5, 10, and 20 mg/mL) in the presence of 4 µM **ARCAM-1** in MEM (Fig. 2C) showed only slight changes in the intensity of absorption, which were also similar to those observed in DI water solutions (Fig. SI1). In fact, the relationship between absorption and **ARCAM-1** concentrations (1, 2, 4, 8, and 16 µM) in the presence of 10 mg/mL BSA in both DI water and MEM exhibited linear plots (Fig. 2D) and a molar extinction coefficient value that was comparable to that in BSA-free solutions. Similar data were obtained for 5 and 20 mg/mL of BSA (Fig. SI2).

### FMR emission spectra

**Figure 3:**
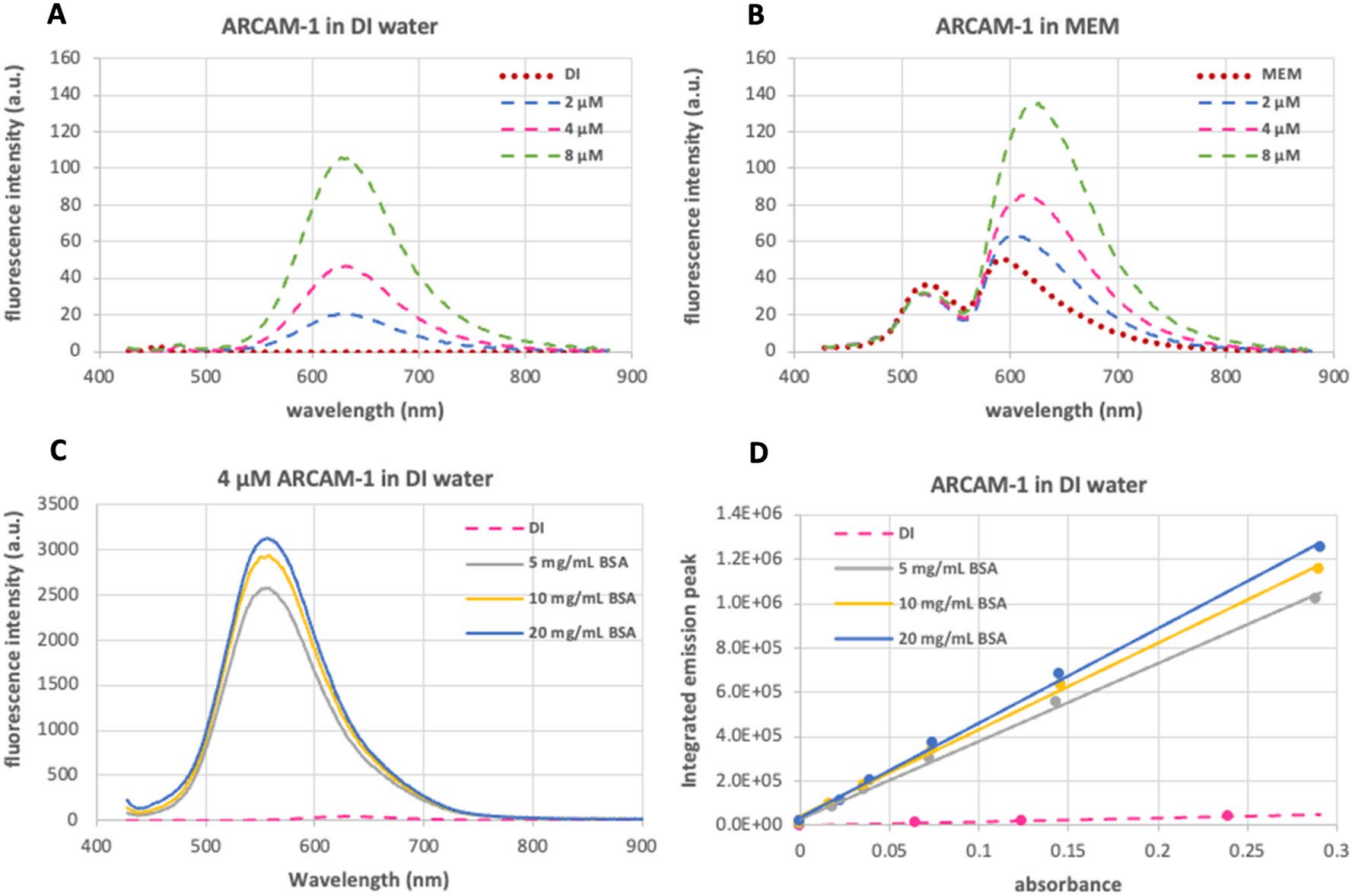
Emission spectra of **ARCAM-1**. Emission is represented with dotted lines for media only, with dashed lines for FMR (different concentrations) and with solid lines for FMR in BSA solutions. Spectral characterization of the FMR was performed at fixed excitation of 410 nm in: (**A**) deionized water (DI), (**B**) MEM, (**C**) 4 µM **ARCAM-1** in presence of different concentrations of BSA (5, 10, and 20 mg/mL) in DI water. (**D**) Calibration curves of integrated emission peak vs absorbance of **ARCAM-1** (1, 2, 4, 8, 16 µM) in DI water with/without BSA.

For the fluorescent emission studies, we utilized a fixed excitation wavelength of 410 nm corresponding to the maximum absorption peak of **ARCAM-1** when in the presence of BSA, a representative protein. In all cases, we observed an emission peak with a maximum emission wavelength (λmax) in the range of 617–630 nm (Table SI1). Specifically, Fig. 3A shows the FMR emission in DI water with a λmax at 629 nm. Similarly, the FMR emission in PBS or MEM showed a peak at λmax = 630 and 617 nm, respectively (Fig. 3B and Fig. SI3). In the latter case, we observed additional emission peaks at λmax = 522 and 585 nm that can be attributed to the intrinsic fluorescence of the medium (Fig. 3B)^24^. In all cases the fluorescence intensity of **ARCAM-1** was found to be concentration-dependent. However, this dependency is negligible since upon protein binding, the FMR emission increases significantly. In fact, the spectrum of **ARCAM-1** (2 µM) in DI water shows two important characteristics. The first characteristic is a hypsochromic shift in the emission (λmax = 555 nm) compared to that of the BSA-free solutions (λmax = 629 nm). The second characteristic is an emission peak that increases with increased concentration of BSA (5–20 mg/mL) and is markedly higher compared to that in the absence of protein (Fig. 3C). These characteristics are attributed to the intrinsic environment-sensing properties of **ARCAM-1** including sensitivity to both polarity (i.e., solvatochromicity)^29^ and viscosity and are both occurring when bound to proteins, as observed previously^26^.

The calibration curves depicting the integrated fluorescence intensity against the absorbance of **ARCAM-1** (Fig. 3D) clearly show that the gradient of the FMR (1, 2, 4, 8 and 16 µM) in DI water was relatively low indicating that the vast majority of the excitation energy is converted to mechanical decay. However, upon binding of **ARCAM-1** to BSA the movement of the FMR becomes restricted, leading to an increased slope. This is evident by the slope of emission versus absorbance of **ARCAM-1** in the presence of 5, 10, and 20 mg/mL of BSA in DI water (Fig. 3D), that shows a significant enhancement, indicating an augmented fluorescence quantum yield with increasing protein concentrations. The lowest concentration of BSA that could be detected using 2 µM of **ARCAM-1** was calculated at 0.012 mg/mL using a fluorescence titration assay (Fig. SI4)^30,31^.

**Figure 4:**
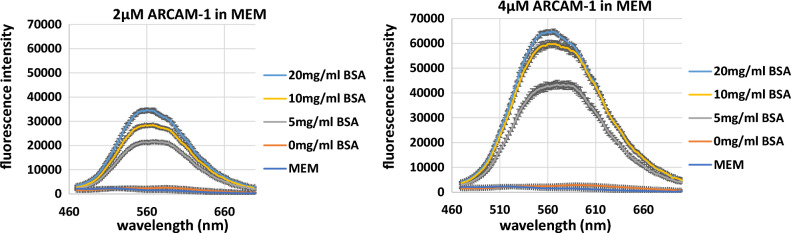
Experimental replicates of **ARCAM-1** in presence of different concentrations of BSA (5, 10, and 20 mg/mL) in MEM. Left panel: **ARCAM-1** at 2 µM. Right panel: **ARCAM-1** at 4 µM. Data collected by microplate reader (Biotek Synergy H1).

These compelling findings inspired us to further explore the utility of **ARCAM-1** for protein quantitation. To enhance its usefulness in biological applications that commonly require small sample volumes, we employed a microplate reader. Additionally, we utilized BSA dissolved in MEM, a commonly used growth medium in cell-based assays. The results paralleled the above findings and clearly showed a concentration-dependent fluorescence emission of **ARCAM-1** (2 and 4 µM) in the presence of increasing concentrations of BSA (5, 10, and 20 mg/mL), as shown in Fig. 4. It is also worth noting that increasing the BSA concentration induces a blue shift in the emission maximum. Specifically, the emission λmax moves from 560 to 540 nm upon increasing BSA concentration from 5 mg/mL to 20 mg/mL. This is attributed to the decrease of polarity that is experienced by the FMR upon binding to the hydrophobic protein pocket.

### FMR emission in the presence of a homogeneous protein (BSA) solution

**Figure 5:**
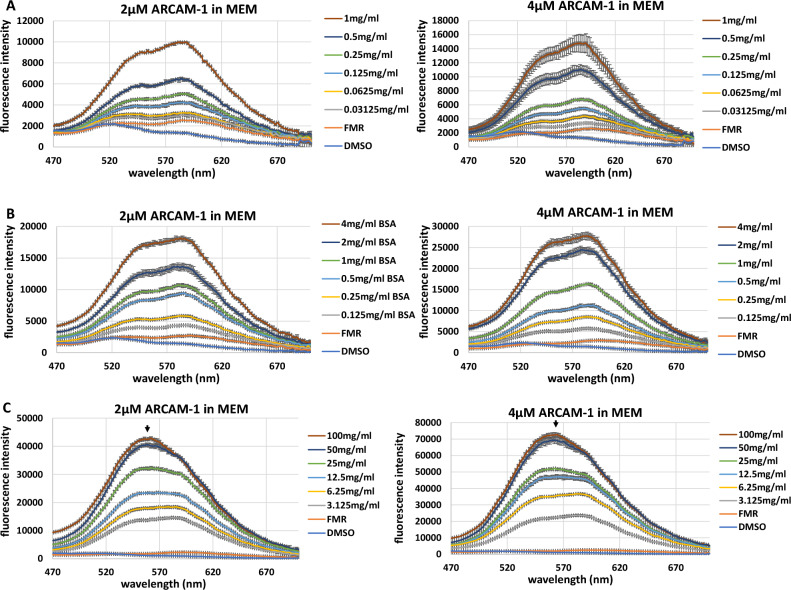
FMR protein concentration resolution (i.e., emission spectra resolution) of BSA: Correlation between FMR emission spectra peak (i.e., quantum yield increase) and protein concentration at (**A**) low-range; 2 µM, left panel, 4 µM, right panel, (**B**) mid-range; 2 µM, left panel, 4 µM, right panel, and (**C**) high-range; 2 µM, left panel, 4 µM, right panel. Standard deviation performed on technical replicates.

**Figure 6:**
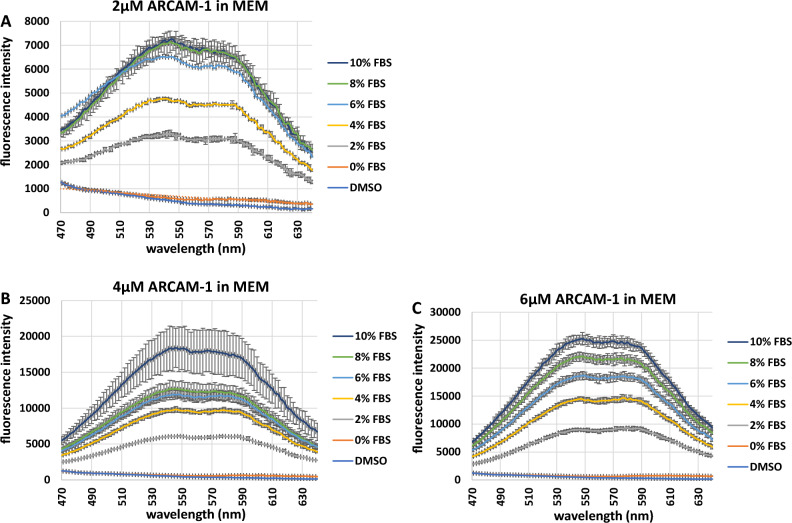
FMR protein concentration resolution (i.e., emission spectra resolution) of FBS: Correlation between FMR emission spectra (i.e., quantum yield increase) and protein concentration (0–10% FBS) at (**A**) 2 µM, (**B**) 4 µM and (**C**) 6 µM. Standard deviation performed on technical replicates.

Encouraged by the above results, we sought to quantify the resolution of FMR emission in the presence of different ranges of protein concentrations. BSA concentrations, arbitrarily defined as low (0 – 1 mg/mL), mid (0 – 4 mg/mL) and high (0 – 200 mg/mL), were made in MEM using serial dilutions. **ARCAM-1** was added to each BSA sample either at a 2 µM or 4 µM final concentration and then samples (in triplicate) were analyzed in a 96-well solid black bottom plate. Spectral analysis was performed using the fixed, 410 nm excitation wavelength, and with a spectral emission window of 470–640 nm. Similarly to Fig. 4, increasing the BSA concentration induces a blue shift in the emission λmax. The emission spectra of the FMR, at both 2 µM and 4 µM, displayed a linear correlation with increasing protein concentration (Fig. 5) and increase in relative fluorescence intensity. In both the low and mid-range protein concentrations at 2 µM and 4 µM there is a clear resolution between protein concentrations (Fig. 5A,B; left and right panel) as dictated by the fluorescence intensity of each of the emission spectral peaks. However, at the high protein concentrations of 50 mg/mL and 100 mg/mL, resolution between spectral peaks decreased slightly (Fig. 5C; left and right panel, black arrows). This is also confirmed in the corresponding linear plots (Fig. SI5). The decrease in resolution may be due to the incomplete saturation of hydrophobic pockets (i.e., unoccupied sites) of BSA protein by FMR. An increase in FMR concentration (e.g., 6 µM or 8 µM) would potentially resolve spectral peaks at higher protein concentrations (Fig. 6C). Our data demonstrate the efficiency of **ARCAM-1** as an effective tool to resolve protein concentrations over a broad range (i.e., 0 – 50 mg/mL) of a homogeneous protein solution.

### FMR emission in the presence of a heterogeneous protein (FBS) solution

To further determine the capabilities of **ARCAM-1** as a tool to quantify protein, we examined FMR’s performance in a heterogeneous protein solution, using fetal bovine serum (FBS). FBS contains more than 80 different proteins (including BSA) at an estimated combined concentration of 37 µg/µL, with each protein having a unique number of hydrophobic pockets^32^. Solutions of increasing percentages (0–10% or 0–3.7 µg/µL protein concentration) of FBS were prepared in MEM. **ARCAM-1** was added to each FBS sample either at a 2 µM, 4 µM or 6 µM final concentration and analysis was performed in triplicates in a 96-well solid black bottom plate. A near linear correlation was observed between emission spectra peak(s) and increasing protein concentration (i.e., increasing percent of FBS) of the 2 µM and 4 µM FMR (Fig. 6A,B); while a linear correlation was found when using 6 µM FMR (Fig. 6C). At FBS > 6% (i.e., higher protein concentrations) there was a slight loss in resolution between emission spectral peaks at both 2 µM and 4 µM FMR (Fig. 6A,B; black arrows). However, at 6 µM FMR (Fig. 6C; red arrow) the emission spectra peak resolution (i.e., protein concentration resolution) displayed marked resolution from 0%—10% FBS with nominal variation of technical replicates (Fig. 6C). The protein concentrations at > 6% FBS are well within the capabilities of **ARCAM-1**, where 2 µM and 4 µM FMR concentrations were optimal for resolving protein concentrations from 0 µg/µL – 4 µg/µL of BSA (Fig. 5B). The lack of peak resolution of previously resolved spectral peaks (Fig. 5B, i.e., mid-range protein concentrations), suggests that this heterogeneous population of proteins presents more hydrophobic pockets as compared to lower concentrations of the homogeneous BSA. This is overcome when presumably saturation of hydrophobic pockets is reached as seen at 6 µM FMR (Fig. 6C). Linear plot data concurs with these findings (Fig. SI6). The combined data unveil **ARCAM-1** to be both amenable to resolving a wide range of protein concentrations and versatile by maintaining a linear correlation between fluorescence intensity (due to constrained molecular rotation while bound non-specifically to protein hydrophobic pockets) and protein concentration of both homogeneous (defined number of potential binding pockets) and heterogeneous (undefined number of binding pockets).

To determine the stability of **ARCAM-1**’s sensing ability over time, one technical replicate from both the 4 µM and 6 µM FMR in FBS was analyzed following a 24-h and 48-h time period (Fig. SI7). Emission spectral resolution for both the 24 and 48-h time points were stable. However, the 24-h and 48-h samples for both the 4 µM and 6 µM FMR decreased in relative fluorescent intensity by ~ 1.7 and 1.3 respectively, in comparison to the day of the experiment (Fig. 6B,C and Fig. SI7). Our data demonstrate the stability of **ARCAM-1** as a tool to quantitate/resolve protein concentrations over a 48-h period.

### Comparison between the Bicinchoninic acid (BCA) assay and FMR for protein quantification of an unknown sample

**Figure 7:**
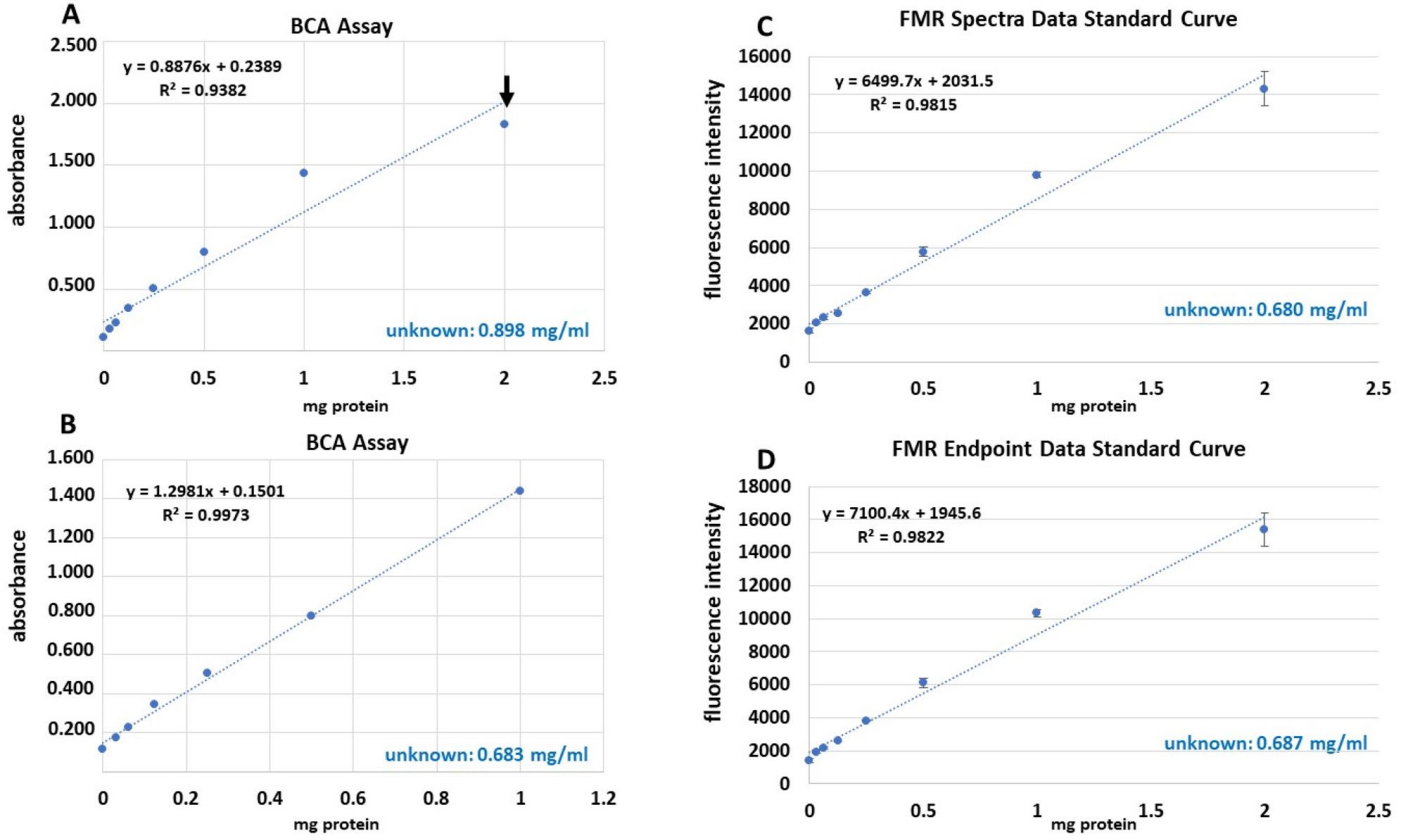
BCA vs. FMR protein concentration analysis of an unknown: (**A**) Bicinchoninic acid protein concentration assay identified unknown protein concentration to be 0.898 mg/mL. (**B**) Out-lying point (**A**; black arrow) was removed and the BCA data replotted, determining unknown protein concentration to be 0.683 mg/mL (**C**) FMR emission spectra data and (**D**) endpoint data determined unknown protein concentration to be 0.680 mg/mL and 0.687 mg/mL respectively.

The BCA assay is a standard technique for quantitation of unknown protein concentrations. In this study, protein standards were made from lyophilized BSA in concentrations ranging from 0 -2 mg/mL. The protein standards and a prepared solution of unknown concentration (*note: unknown was tested in a blind manner) were loaded into a 96-well plate. Working reagents were added according to manufacturer’s protocol (Pierce™ BCA Protein Assay Kit, Thermo Fisher Scientific). The absorbance of the multi-well plate was read at 562 nm. A standard curve and linear regression (best fit) were derived from the absorbance data (Fig. 7). Data interpolation determined the unknown solution to have a protein concentration of 0.898 mg/mL (Fig. 7A).

A suspected out-lying absorbance point (exhibited lower R^2^ value; data not shown) was removed from the BCA data (Fig. 7A, arrow) and a new standard curve and linear regression (best fit) was constructed (Fig. 7B). From this plot, the concentration of the unknown was found to be 0.683 mg/mL (Fig. 7B).

Using the same standards prepared for the BCA assay and unknown, in a side-by-side experiment the performance of the FMR in identifying the protein concentration of an unknown sample was tested. The FMR was added to the protein standards at a 2 µM concentration and then loaded into a 96-well solid black bottom plate along with the unknown, in replicates. Both emission spectra data (maximum fluorescence intensity value taken from emission spectra between 450–700 nm), and endpoint data (fluorescence intensity value at 590 nm) were collected with an excitation window set to 410 nm for both readings. Emission spectra and endpoint data were plotted against the BSA concentrations to construct two standard curves and linear regression best fits (Fig. 7C,D). The equations derived for the linear regression (best fit) allowed for interpolation of the unknown sample. The best fit measurement from both emission spectra and endpoint data were comparable. The concentration of the unknown using the FMR was found to be 0.680 mg/mL using the emission spectra data and 0.687 mg/mL emission endpoint data (Fig. 7C,D) respectively.

The unknown for both the BCA and FMR assay was made by taking 0.3 mL from the 2 mg/mL BSA protein standard and diluting with 0.6 mL of deionized water. This provided a final concentration of 0.666 mg/mL that was used in the side-by-side comparison. The BCA based quantitation had a 34% (Fig. 7A) and 2.5% (outlier removed; Fig. 7B) error rate whereas the emission spectra and endpoint FMR data had a 2.2% and 3.2% error rate, respectively (Fig. 7C,D).

### Evaluation of FMR for in situ flow measurements

**Figure 8:**
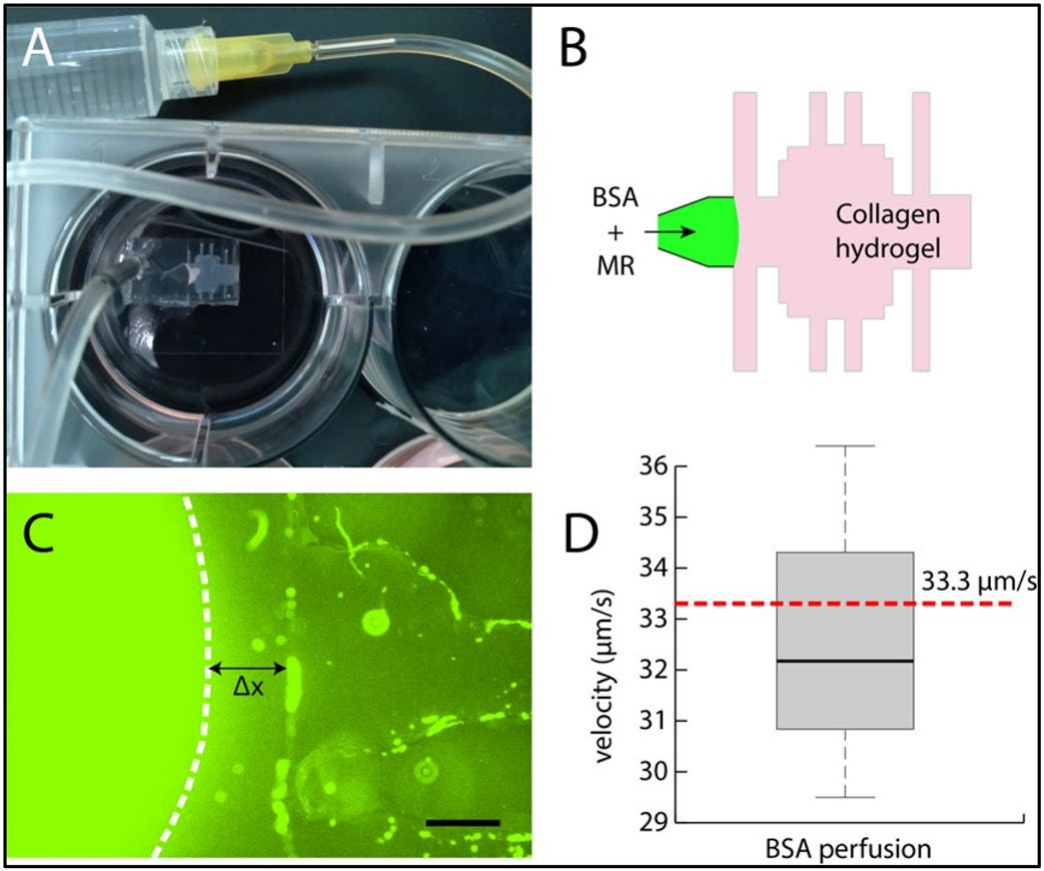
Microfluidic flow validation: (**A**) Photograph of the microfluidic device perfused with BSA and molecular rotors. (**B**) Schematic of the collagen hydrogel within the device. (**C**) Fluorescent front tracked with an epifluorescent microscope, scale = 400 microns. (**D**) Quantification of velocity and comparison with expected value.

Findings from extrinsic analyses, specifically the ability to accurately resolve a broad spectrum of protein concentrations, prompted evaluation of the FMR as a probe to visualize transport within a protein hydrogel. Tracking interstitial fluid flow with fluorescently labeled microparticles is often not feasible in fibrous matrices due to the pores constraining the particles. However, the protein-sensing capabilities of **ARCAM-1** can be used to fluorescently label soluble proteins convecting through a hydrogel. To validate this application of FMR, a collagen type I hydrogel-filled microfluidic chamber was perfused with a bolus of protein (10 µg/mL BSA) in the presence of FMR (2 µM). A photograph of the device and schematic of the experiment are provided in Fig. 8A,B. The protein-FMR solution was administered using a syringe pump with a flow rate set at 10 µL/min with real-time fluorescent images taken every 0.5 s for in situ evaluation. This flow rate was chosen to assure that convective transport dominated any diffusive effects. Using a previously recorded measurement of albumin diffusivity in collagen hydrogels^33^, the Peclet number for this flow was calculated to be approximately 1000, verifying that the rate of the interstitial fluid flow could be quantified by tracking the fluorescent front. Measurements of the distance traveled by the fluorescent front (labeled as Δx in Fig. 8C) were taken in three locations and the average and standard deviation of interstitial flow velocity was determined based on the time interval between measurements. Figure 8D indicates that the measured velocity values were within one standard deviation of the expected velocity of 33.3 µm/s based on the 10 µL/min volumetric flow rate divided by a cross-sectional area of the collagen gel, validating the accuracy of the **ARCAM-1**-labeled measurement.

### Evaluation of cell compatibility following ARCAM-1 exposure

**Figure 9:**
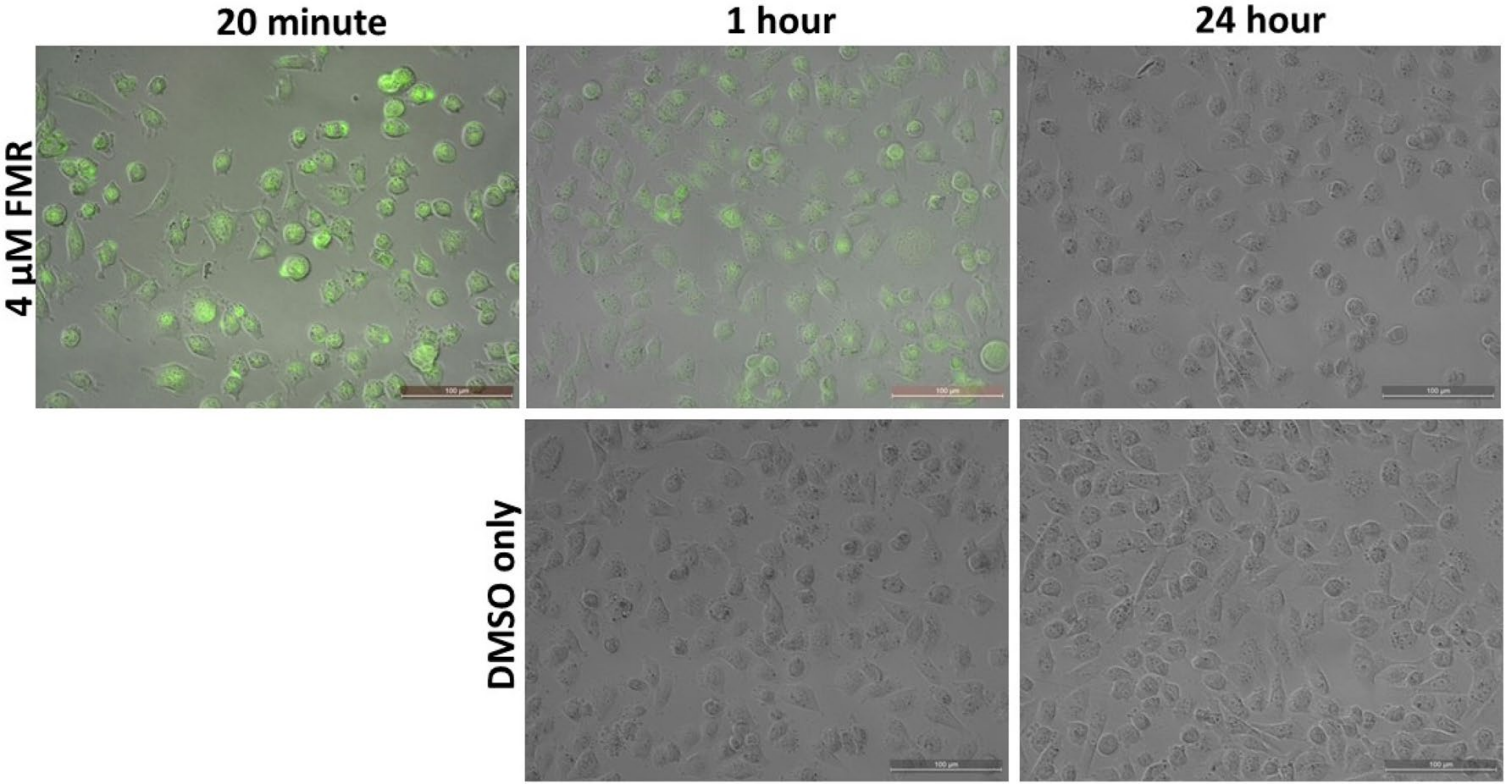
Cell compatibility analysis: MDA MB 231 cells with 4 µM FMR concentration at 20-min, 1-h and 24-h; 20X, 100 µm bar.

Protein quantitation is often necessary for cellular extracts or secreted factors and can be performed exogenously via direct measurement (e.g., UV absorption), protein assays that are compared to standards (e.g., BCA, Lowry, etc.) or measurement of a specific protein or proteins of interest (e.g., western blot analysis, enzyme-linked immunosorbent assay, mass spectrometry)^6,34^. However, to our knowledge, none of these assays can be used within cellular systems as in situ protein quantitation probes over extended periods of time. To determine if **ARCAM-1** could be used in cell-based assays, evaluation of cell compatibility was performed on the MDA MB 231 invasive breast cancer cell line (ATCC catalogue #HTB-26). MDA MB 231 cells were grown in a 24-well plate and treated with either 1 µM, 2 µM or 4 µM of FMR and analyzed on a fluorescent microscope at 20 min, 1-h and 24-h to observe cell viability following **ARCAM-1** exposure (Fig. 9 and Fig. SI8A). Following a 20-min exposure at 1 µM, 2 µM and 4 µM, the FMR is distributed throughout the cytoplasm of the cells (Fig. 9 and Fig. SI8A; 20 min, 1 µM, 2 µM or 4 µM of FMR) with perinuclear and nuclear localization. FMR perinuclear and nuclear localization becomes more distinct for all three concentrations following a 1-h exposure (Fig. SI8B). At 24 h, cellular FMR was no longer visible. Significantly, no cytotoxic effects were seen for all FMR concentrations and time exposures (Fig. 9 and Fig. SI8A,B; experimental compared to DMSO only).

## Conclusion

This study demonstrates for the first time the efficiency of **ARCAM-1,** a representative fluorescent molecular rotor (FMR), to quantitate both homogeneous and heterogeneous protein solutions. This FMR can determine unknown protein concentrations in a manner that is consistent and comparable to other currently used protein concentration assays. Moreover, it offers significant advantages over the current assays due to its application for in situ measurements without affecting cell viability. Specifically, **ARCAM-1** can be used in various experimental systems for real-time protein quantitation of either homogeneous or heterogeneous protein solutions over a broad range of protein concentrations. Moreover, it does not alter protein function, requires no additional reagents for the measurements and is compatible with use in cell systems. The unique FMR-hydrophobic interactions, that result in a quantum yield increase in fluorescence, provide an efficient method of protein quantitation. Protein concentration is efficiently resolved by minor adjustments of FMR concentration. Excess FMR does not interfere with protein concentration evaluation as only FMR-hydrophobic pocket interactions result in quantum yield increases in fluorescence. Most importantly, this FMR has the potential to function as an environmental indicator of in situ protein crowding within cells and soluble factor gradient evaluation. This work suggests that the FMR protein quantitation assay may be advantageous over currently used colorimetric assays since: (i) it does not rely on chemical conversion for results (i.e., no chelation), (ii) it is not a terminal assay (i.e., it does not interfere with cell viability or protein activity), (iii) it can be performed in situ (within unique experimental parameters e.g., microfluidic device), (iv) it is sensitive over a broad spectrum of protein concentrations (homogeneous and heterogeneous populations), and (v) it allows for sample measurement over extended experimental periods.

## Materials and methods

### Chemical synthesis of ARCAM-1

**Scheme 1:**
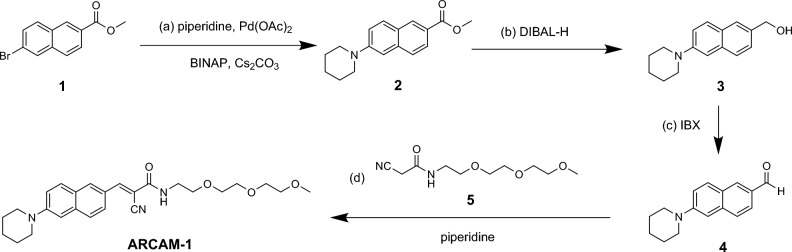
Synthesis of **ARCAM-1**. *Reagents and conditions:* (**a**) 1.0 eq. **1**, 1.13 eq piperidine, 0.15 eq BINAP, 0.02 eq Pd(OAc)_2_, 2.0 eq cesium carbonate, toluene, overnight, 100 °C, 79%; (**b**) 1.0 eq. **2**, 5.0 eq. 1.0 M DIBAL-H in hexane, THF, overnight, 25 °C, 95%; (**c**) 1.0 eq. **3**, 3.0 eq IBX, DCM, overnight, 0–25 °C, 88%; (**d**) 1.0 eq. **4**, 1.2 eq. **5**, 0.2 eq piperidine, THF, overnight, 50 °C, 62%.

This compound was synthesized by modification of a previously reported synthesis that improved the overall purification/yield^26,35^. Commercially available methyl-6-bromo-2-naphthoate (**1**) underwent Buchwald coupling with piperidine to form adduct **2** that was converted to aldehyde **4** via reduction with DIBAL-H and oxidation with IBX (3 steps, 66% combined yield). The 2-cyano-*N*-(2-(2-(2-methoxyethoxy)ethoxy)ethyl)acetamide (**5**) was synthesized following our previous procedure^26^. Finally, Knoevenagel condensation of aldehyde **4** with **5** under piperidine catalysis produced the desired **ARCAM-1**, in 62% isolated yield (Scheme 1). Additional information on the commercial sources of all reagents, detailed experimental methods and spectroscopic characterization (^1^H and ^13^C NMR) for all synthetic intermediates can be found in the Supporting Information.

### FMR stock solution preparation

**ARCAM-1** was solubilized in dimethyl sulfoxide (DMSO) (Fisher Scientific catalog #97,063–136) for a final concentration of 10 mM and stored at – 20 °C in 20 µL aliquots.

### Protein sample preparation

Homogeneous solutions: Lyophilized bovine serum albumin (BSA) (Fisher Scientific catalogue #BP671-1) protein samples from 0 – 100 mg/mL concentrations were prepared in Minimal Essential Media (MEM) (Fisher Scientific; Gibco™ catalogue #11–095-072). Heterogeneous solutions: Fetal bovine serum (FBS) (Fisher Scientific; Gibco™ catalogue #10–438-026) samples were prepared in MEM solutions from 0%—10% concentrations.

### FMR absorption spectra

**ARCAM-1** spectral data were collected using a UV–Vis Spectrophotometer (Duetta™ Spectrometer). Spectral measurements were performed at ambient temperature and the absorbance spectra were obtained from 300–500 nm in 2 nm wavelength increments, 0.05 s integration time in 10 × 10 mm inner size cuvettes. **ARCAM-1** was dissolved in different aqueous solvents (DI water, PBS pH 7.2, and MEM) in final concentrations of 1–16 µM. The solvents were purchased from the following sources; PBS: Fisher Scientific; Gibco^TM^catalogue #20–012-027.

### FMR emission spectra

The fluorescence emission spectra of **ARCAM-1** were assessed using spectrophotometer (Duetta™ Spectrometer) and microplate readers (Biotek Synergy H1 Hybrid). For both instruments, excitation was set at 410 nm and emission spectra data were collected from 450 – 900 nm or 450 – 700 nm in 2 nm wavelength increments. Duetta™ Spectrometer: 0.05 s integration time in 10 × 10 mm inner size cuvette. Biotek Synergy H1 Hybrid: The z-height (top down) was set at 7 mm.

### Determination of the limit of protein detection of FMR

The lowest amount of BSA that could be detected with 2 µM of **ARCAM-1** was calculated using the equation LOD = 3.3*σ/S where σ is the standard deviation of the fluorescence intensity of the pure probe and S is the slope of the calibration curve. To calculate σ, we measured the fluorescence intensity of the probe for 10 times. The slope was calculated from the fluorescence intensity of the probe in the presence of increasing concentrations of BSA in triplicate^31^.

### Bicinchoninic Acid (BCA)-based protein quantitation assay

Bovine serum albumin standards from 0 µg/mL to 2000 µg/mL were prepared in milli Q ultrapure water. Following the micro BCA™ protein assay kit protocol, in a clear 96-well plate the working reagent was added to both standards and unknown in an 8:1 ratio. A Biotek Synergy H1 Hybrid Multi-Mode Reader was used to read absorbance of samples at 562 nm. The unknown for the BCA assay was made by taking 0.3 mL from the 2 mg/mL BSA protein standard and diluting with 0.6 mL of deionized water.

### FMR-based protein quantitation assay

Bovine serum albumin standards from 0 µg/mL – 2000 µg/mL were prepared in milli Q ultrapure water. FMR at a final concentration of 2 µM was added to each standard and unknown sample in a 96-well solid black bottom plate. Fluorescent measurements were taken on the Synergy H1 plate reader with excitation set to 410 nm. Standard curves were plotted using both emission endpoint data where fluorescence intensity value at 590 nm is used and maximum data where the maximum fluorescence value for each sample is used regardless of emission wavelength value. The unknown for the FMR assay was made by taking 0.3 mL from the 2 mg/mL BSA protein standard and diluting with 0.6 mL of deionized water.

### Microfluidic interstitial flow assay

Polydimethylsiloxane (PDMS)-based microfluidic chambers were filled with a 2 mg/mL collagen type I hydrogel, consisting of 0.1 M NaOH, 10X phosphate buffered saline (PBS), and 4 mg/mL collagen solubilized in 0.02 M acetic acid. The gels were polymerized at 37 °C for at least one hour prior to experiments. FMR was added to a final 2 μM concentration in PBS containing 10 μg/mL of BSA. A syringe pump was used to perfuse the hydrogel at a flow rate of 10 μL/min on the stage of an epifluorescent microscope (Nikon Ti-E) and images were taken every 0.5-s with a 488-nm emission filter. The dynamics of the fluorescent front were measured in three locations to determine the average and standard deviation of the interstitial flow velocity and were compared to the expected value based on the flow rate and cross-sectional area of the device (1-mm × 5-mm).

### Cell compatibility analysis

MDA MB 231 cells (ATCC catalogue #HTB-26) were seeded in a 24-well plate with MEM, 10% fetal bovine serum and antibiotics. Cells were maintained in humidified air with 5% CO_2_ at 37 °C. Upon reaching ~ 85% confluency, 1 µM, 2 µM and 4 µM concentration of FMR was added to individual wells and analyzed using a Leica DM IRE2 Inverted Fluorescence DIC Polarization Phase Contrast Microscope at 20 min, 1-h, and 24-h.

### Supplementary Information

Supplementary Information.

## Data Availability

The raw data sets generated will be made available upon request to the corresponding authors.
