# Supplementary material for: Fluorescent molecular rotors as versatile in situ sensors for protein quantitation
Source: Sci Rep. 2023 Nov 22;13:20529. doi: 10.1038/s41598-023-46571-5 (PMC10665405; doi:10.1038/s41598-023-46571-5)
Supplement: Supplementary file 1 — Supplementary Information. [file 41598_2023_46571_MOESM1_ESM.docx]

**Fluorescent molecular rotors as versatile *in situ* sensors for protein quantitation**

**Kevin Daus^1,Ϯ^, Sorachat Tharamak^2,3,Ϯ^, Wanchai Pluempanupat^3^, Peter A. Galie^4^, Maria A. Theodoraki^5^, Emmanuel A. Theodorakis^2,*^** **and Mary L. Alpaugh^1,*^**

*^1^Department of Biological and Biomedical Sciences, Rowan University, 201 Mullica Hill Rd, Glassboro, New Jersey 08028, USA*

*^2^Department of Chemistry and Biochemistry,* *University of California, San Diego, 9500 Gilman Drive, La Jolla, California, 92093-0358, USA*

*^3^Department of Chemistry and Center of Excellence for Innovation in Chemistry, Special Research Unit for Advanced Magnetic Resonance, Faculty of Science, Kasetsart University, Bangkok, 10900, Thailand*

*^4^Department of Biomedical Engineering, Rowan University, Glassboro, NJ 08028, USA*

*^5^Department of Biology, Arcadia University, 450 S. Easton Rd, Glenside, PA 19038, USA*

**SUPPORTING INFORMATION**

General chemistry procedures S2

Abbreviations of chemical names, Commercial sources/catalogue number nomenclature S2

Experimental procedures for the chemical synthesis of **ARCAM-1** S2

Table SI1. Spectroscopic characteristics of **ARCAM-1** in different aqueous solutions. S4

Figure SI1. Absorbance spectra of **ARCAM-1** in presence of different concentrations of BSA. S4

Figure SI2. Molar extinction coefficient of **ARCAM-1** in presence of BSA. S5

Figure SI3. Emission spectrum of **ARCAM-1** (2, 4, and 8 µM) in PBS. S5

Figure SI4. Measuring the Limit of Detection (LOD) of **ARCAM-1** (2 µM) of BSA in DI water S6

Figure SI5. Linear plots of **ARCAM-1** in present of different concentrations of BSA. S7

Figure SI6. Linear plot of **ARCAM-1** in present of different percentages of FBS S7

Figure SI7. Duration of FMR sensitivity. S8

Figure SI8. Cell compatibility analysis. S9

NMR Spectra of **ARCAM-1** and synthetic intermediates. S10

**General chemistry procedures.** Unless indicated otherwise, all commercially available reagents and anhydrous solvents were purchased at the highest commercial quality and were used as received without further purification. All non-aqueous reactions were carried out under argon atmosphere using dry glassware that had been flame-dried under a stream of argon unless otherwise noted. Anhydrous dichloromethane (DCM) and tetrahydrofuran (THF) were obtained by passing commercially available pre-dried, oxygen-free formulations through activated alumina columns. Flash column chromatography was performed on silica gel (Silicycle Inc., 230-400 mesh) using solvent mixtures of increasing polarity. The progress of all the reactions was monitored by thin-layer chromatography (TLC) using glass plates precoated with silica gel-60 F_254_ to a thickness of 0.25 mm (Silicycle Inc.). ^1^H and ^13^C NMR spectra were recorded on either 300 MHz (Bruker), 400 MHz and 500 MHz JEOL instrument. CDCl_3_ was treated with flame dried K_2_CO_3_, chemical shifts (δ) are quoted in parts per million (ppm) referenced to the appropriate residual solvent peak or TMS internal reference (CDCl_3_ -TMS), with the abbreviations s, br s, d, t, q, m, qd and dt denoting singlet, broad singlet, doublet, triplet, quartet, multiplet, quartet of doublet and doublet of triplets respectively. *J* = coupling constants given in Hertz (Hz).

**Abbreviations of chemical names Commercial sources/catalogue number**

BINAP = (±)-2,2′-bis(diphenylphosphino)-1,1′-binaphthyl Sigma-Aldrich (481084)

Cs_2_CO_3_ = cesium carbonate Thermo Fisher Scientific (AA1288706)

DCM = dichloromethane Thermo Fisher Scientific (D150-4)

DIBAL-H = diisobutylaluminum hydride Sigma-Aldrich (190306)

EtOAc = ethyl acetate Thermo Fisher Scientific (E145-20)

IBX = 2-Iodoxybenzoic acid Sigma-Aldrich (661384)

Pd(OAc)_2_ = palladium(II) acetate Sigma-Aldrich (520764)

THF = tetrahydrofuran Thermo Fisher Scientific (02-004-427)

**Experimental procedures for the chemical synthesis of ARCAM-1**

**Methyl 6-(piperidin-1-yl)-2-naphthoate (2)**: To a solution of anhydrous, degassed toluene (8.0 mL) was added palladium acetate (17.0 mg, 0.02 mmol, 0.02 equiv.), racemic BINAP (35 mg, 0.06 mmol, 0.15 equiv), and cesium carbonate (2.5 g, 7.7 mmol, 2 equiv.). The heterogenous mixture was allowed to stir for 15 min at 100°C. After cooling to room temperature, piperidine (0.52 mL, 4.5 mmol, 1.125 equiv.) and **1** (1.00 g, 3.8 mmol, 1.0 equiv.) were added, and the reaction was allowed to stir overnight at 100°C. Following completion, the reaction was poured into sodium bicarbonate and extracted with EtOAc (3 x 50.0 mL). The organic layer was washed with brine, dried with MgSO_4_, concentrated under reduced pressure, and purified via flash chromatography (0-10% EtOAc/hexanes) to provide **2** as a white solid (0.813 g, 79%)**:** R_f_ = 0.40 (1:1 hexanes: DCM); R_f_ = 0.40 (1:1 hexanes: DCM); ^1^H NMR (500 MHz, CDCl_3_): δ 8.45 (s, 1H), 7.95 (dd, J = 8.6, 1.7 Hz, 1H), 7.78 (d, J = 9.1 Hz, 1H), 7.65 (d, J = 8.7 Hz, 1H), 7.30 (dd, J = 9.1, 2.5 Hz, 1H), 7.08 (s, 1H), 3.33 (br s, 4H), 1.82 – 1.60 (m, 6H); ^13^C NMR (125 MHz, CDCl_3_): δ 167.72, 151.63, 137.49, 130.82, 130.23, 126.73, 126.56, 125.83, 124.19, 119.90 109.08, 52.07, 50.12, 25.77, 24.47.

**(6-(piperidin-1-yl)naphthalen-2-yl)methanol (3):** To a solution of 1.0 M DIBAL-H in hexanes (4.71 mL, 4.71 mmol, 5.0 equiv.) under argon at 0°C was added a solution of **2** (0.254 g, 0.94 mmol, 1.0 equiv.) in anhydrous THF (3 mL), dropwise. The reaction was warmed to room temperature and allowed to stir overnight. Upon completion, the reaction was cooled to 0°C and MeOH was added dropwise until bubbling ceased, followed by the addition of saturated sodium potassium tartrate to create a jelly-like solution that was stirred vigorously until the layers separated. The reaction was then extracted with EtOAc (3 x 20.0 mL), washed with ammonium chloride and brine, dried with MgSO_4_, concentrated under reduced pressure, then purified via flash chromatography (0-40% EtOAc/hexanes) to provide **4** as a light pink solid (0.215 g, 95%). R_f_ = 0.20 (7:3 hexanes: EtOAc); R_f_ = 0.20 (7:3 hexanes: EtOAc); ^1^H NMR (300 MHz, CDCl_3_) δ 7.69 (d, *J* = 3.4 Hz, 1H), 7.66 (d, *J* = 2.2 Hz, 2H), 7.39 (dd, *J* = 8.3, 2.0 Hz, 1H), 7.29 (dd, *J* = 9.0, 2.5 Hz, 1H), 7.12 (s, 1H), 4.79 (s, 2H), 3.30 – 3.22 (m, 4H), 1.84 – 1.70 (m, 4H), 1.64 – 1.63 (m, 2H);^13^C NMR (125 MHz, CDCl_3_): δ 150.19, 135.71, 134.29, 128.58, 128.21, 127.20, 125.77, 125.48, 120.50, 110.47, 65.68, 51.13, 25.92, 24.41.

**6-(piperidin-1-yl)-2-naphthaldehyde (4):** To a solution of **3** (0.215 g, 0.89 mmol, 1.0 equiv.) in anhydrous DCM (9.0 mL) at 0°C was added IBX (0.76 g, 2.7 mmol, 3.0 equiv.), and the solution was allowed to stir for 30 min. The reaction was then warmed to room temperature and stirred overnight. Following completion of the reaction, aq. NaHCO_3_ (10.0 mL) was added, and the crude product was extracted with EtOAc (3 x 10.0 mL), washed with brine, dried with MgSO_4_, concentrated under reduced pressure, then purified via flash chromatography (0-12% EtOAc/hexanes) to provide **4** as a white solid (0.187 g, 88%). R_f_ = 0.56 (7:3 hexanes: EtOAc); ^1^H NMR (500 MHz, CDCl_3_): δ 9.97 (s, 1H), 8.04 (s, 1H), 7.80 (d, J = 9.2 Hz, 1H), 7.71 (d, J = 9.2 Hz, 1H), 7.61 (d, J = 8.6 Hz, 1H), 7.23 (dd, J = 9.1, 2.5 Hz, 1H), 7.01 (d, J = 2.4 Hz, 1H), 3.31 – 3.29 (m, 4H), 1.70 – 1.66 (m, 4H), 1.62 – 1.58 (m, 2H); ^13^C NMR (125 MHz, CDCl_3_): δ 191.72, 151.80, 138.42, 134.29, 131.18, 130.33, 127.12, 126.16, 123.20, 119.32, 108.61, 49.38, 25.43, 24.22.

**(*E*)-2-cyano-*N*-(2-(2-(2-methoxyethoxy)ethoxy)-ethyl)-3-(6-(piperidin-1-yl)naphthalen-2-yl)acrylamide (ARCAM-1):** To a solution of **4** (76.0 mg, 0.32 mmol, 1.0 equiv.) and **5** (88.4 mg, 0.38 mmol, 1.2 equiv.) in anhydrous THF (3 mL) was added piperidine (6.3 µL, 0.064 mmol, 0.2 equiv.). The mixture was heated to 50 ̊C for 21 hours. Following completion, the reaction was concentrated under reduced pressure, then purified via flash chromatography (10-30% EtOAc/hexanes) to provide **ARCAM-1.** This compound was precipitated under DCM/hexane conditions to form an orange-yellow powder (88 mg, 62%). R_f_ = 0.15 (1:3 hexanes: EtOAc); ^1^H NMR (400 MHz, CDCl_3_) δ 8.37 (s, 1H), 8.16 (s, 1H), 8.05 (dd, *J* = 8.8, 1.7 Hz, 1H), 7.75 (d, *J* = 9.2 Hz, 1H), 7.65 (d, *J* = 8.8 Hz, 1H), 7.29 (dd, *J* = 9.2, 2.4 Hz, 1H), 7.05 (br s, 1H), 6.84 (br s, 1H), 3.72 – 3.61 (m, 10H), 3.61 – 3.54 (m, 2H), 3.40 – 3.35 (m, 7H), 1.79 – 1.69 (m, 4H) 1.69 – 1.62 (m, 2H). ^13^C NMR (101 MHz, CDCl_3_) δ 161.35, 153.17, 151.89, 137.44, 133.94, 130.50, 127.37, 126.78, 126.23, 125.88, 119.64, 117.98, 108.77, 100.62, 72.06, 70.75, 70.71, 70.63, 69.56, 59.17, 49.70, 40.33, 25.66, 24.47.

**Table SI1.** Spectroscopic characteristics of **ARCAM-1** in different aqueous solutions.^a^

| Solvents | λ_abs_ [nm] | λ_em_ [nm] | Stokes shift [nm] | ε [M^-1^cm^-1^] |
| --- | --- | --- | --- | --- |
| DI water | 390 ± 1 | 629 ± 3 | 239 | 1.9 × 10^4^ |
| PBS (pH 7.2) | 390 ± 1 | 630 ± 2 | 240 | 1.9 × 10^4^ |
| MEM | 372 ± 9 | 617 ± 13 | 245 | 1.9 × 10^4^ |
| DI water with BSA | 410 ± 4 | 555 ± 3 | 145 | 1.8 × 10^4^ |
| MEM with BSA | 409 ± 4 | 560^b^ | 151 | 1.9 × 10^4^ |

^a^  Unless otherwise noted, all measurements were performed in a Duetta^TM^ Spectrometer

^b^ Data collected by Biotek Synergy H1 microplate reader.

**
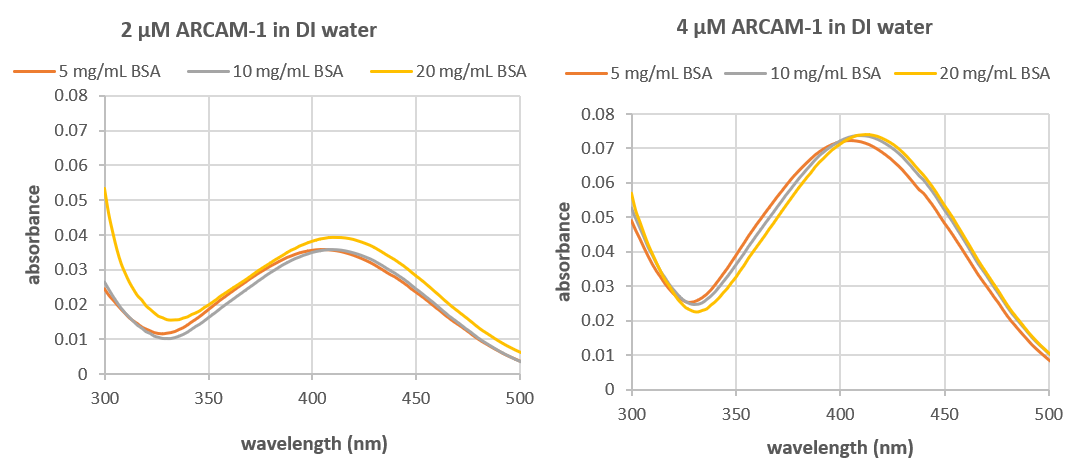
**

**Figure SI1.** Absorbance spectra of **ARCAM-1** in presence of different concentrations of BSA (5, 10, and 20 mg/mL) in DI water using **ARCAM-1** at 2 µM (left panel) and 4 µM (right panel).


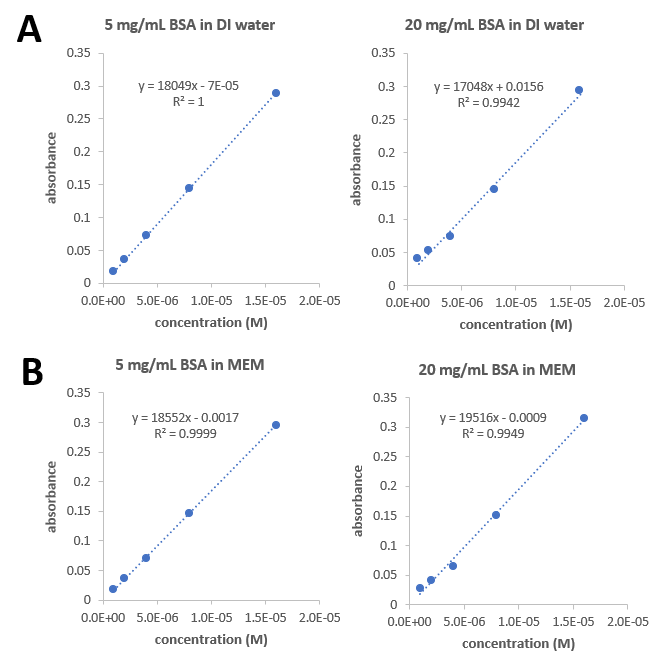


**Figure SI2.** Molar extinction coefficient: **A; left panel)** ARCAM-1 in presence of 5mg/mL BSA in DI water. **A; right panel)** ARCAM-1 in presence of BSA 20 mg/mL in DI water. **B; left panel)** ARCAM-1 in presence of 5 mg/mL BSA in MEM and **B; right panel)** ARCAM-1 in presence of BSA 20 mg/mL in MEM.

**Figure SI3.** **Emission spectrum of ARCAM-1 (2, 4, and 8 µM) in PBS**

**
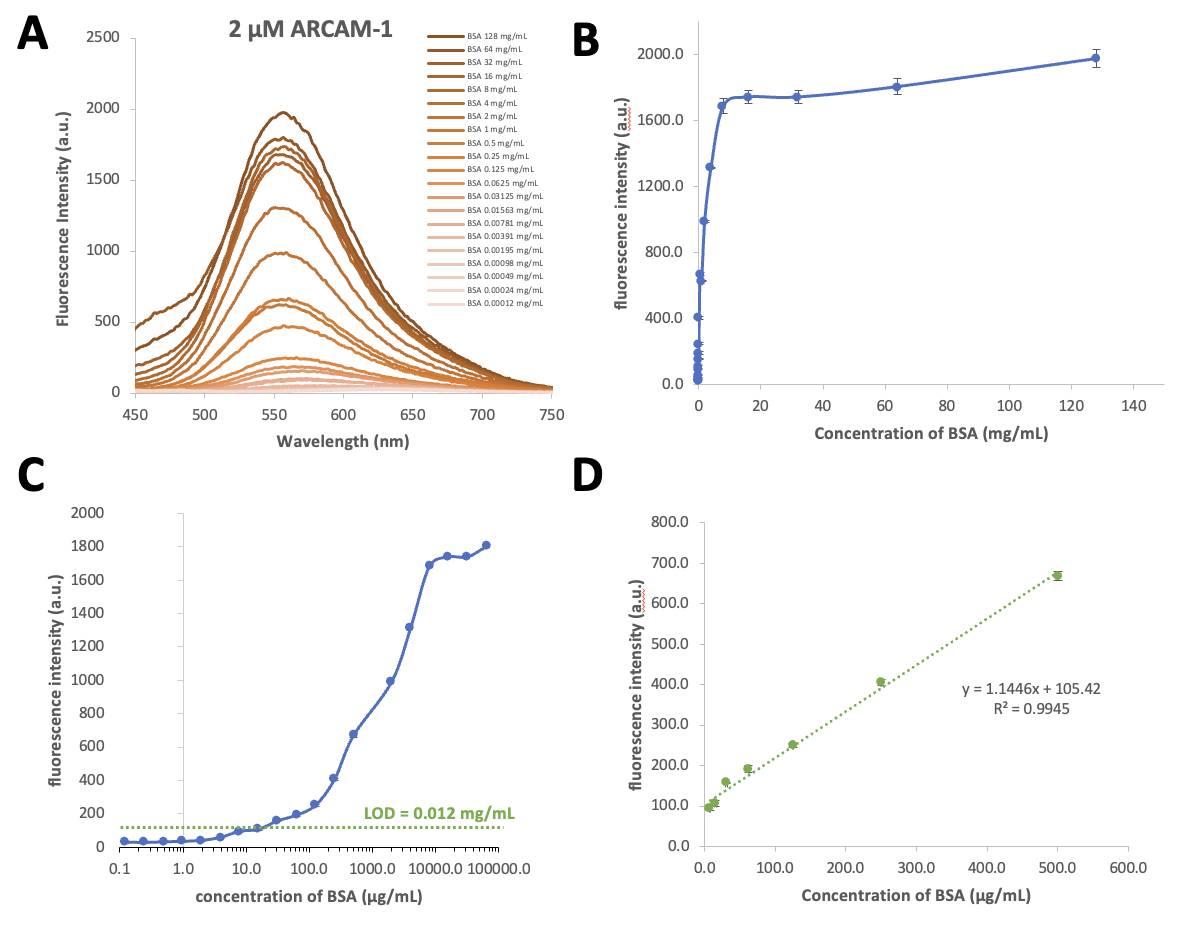
**

**Figure SI4.** **Determination of the limit of detection (LOD) of BSA in the presence of ARCAM-1.** A) Emission spectra of **ARCAM-1** (2 µM) in presence of increasing concentrations of BSA (0.00012-128 mg/mL) in DI water. B) Response of fluorescence intensity versus BSA concentrations. C) Calibration curve of fluorescence intensity versus increasing BSA concentrations in logarithmic scale. D) Calibration curve (linear fit) of fluorescence intensity versus BSA concentrations (0.0078-0.5 mg/mL).


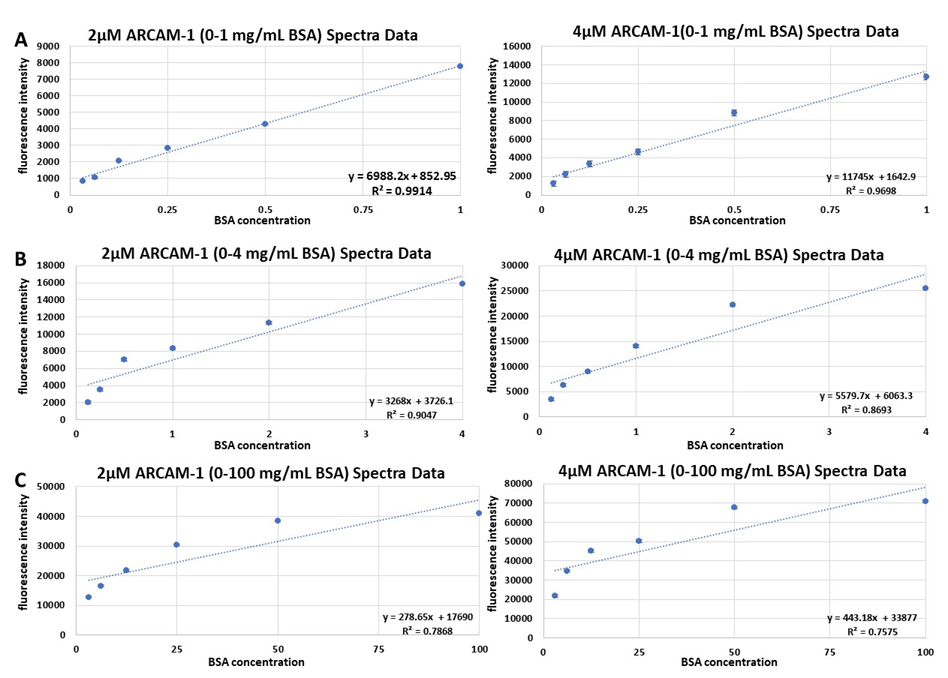


**SI5. Linear plot of fluorescence intensity vs. low, mid and high BSA concentrations:** A) low-range BSA concentrations; 2 µM, left panel, 4 µM, right panel, B) mid-range BSA concentrations; 2 µM, left panel, 4 µM, right panel, and C) high-range BSA concentrations; 2 µM, left panel, 4 µM, right panel.

: A) low-range BSA concentrations; 2µM, left panel, 4µM, right panel, B) mid-range BSA concentrations; 2µM, left panel, 4µM, right panel, and C) high-range BSA concentrations; 2µM, left panel, 4µM, right panel.


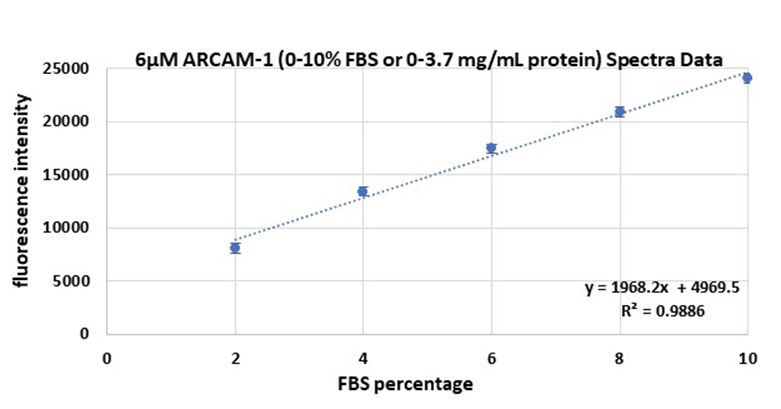


**SI6. Linear plot of fluorescence intensity vs. 0 – 10% FBS**: Linear plot of percent FBS (or 0 – 3.7 mg/mL total protein) vs. fluorescence intensity.

**Figure SI7. Duration of FMR sensitivity:** Emission spectra resolution of **A)** 4 µM and 6 µM FMR post 24-hr period and **B)** 4 µM and 6 µM FMR post 48-hr period.


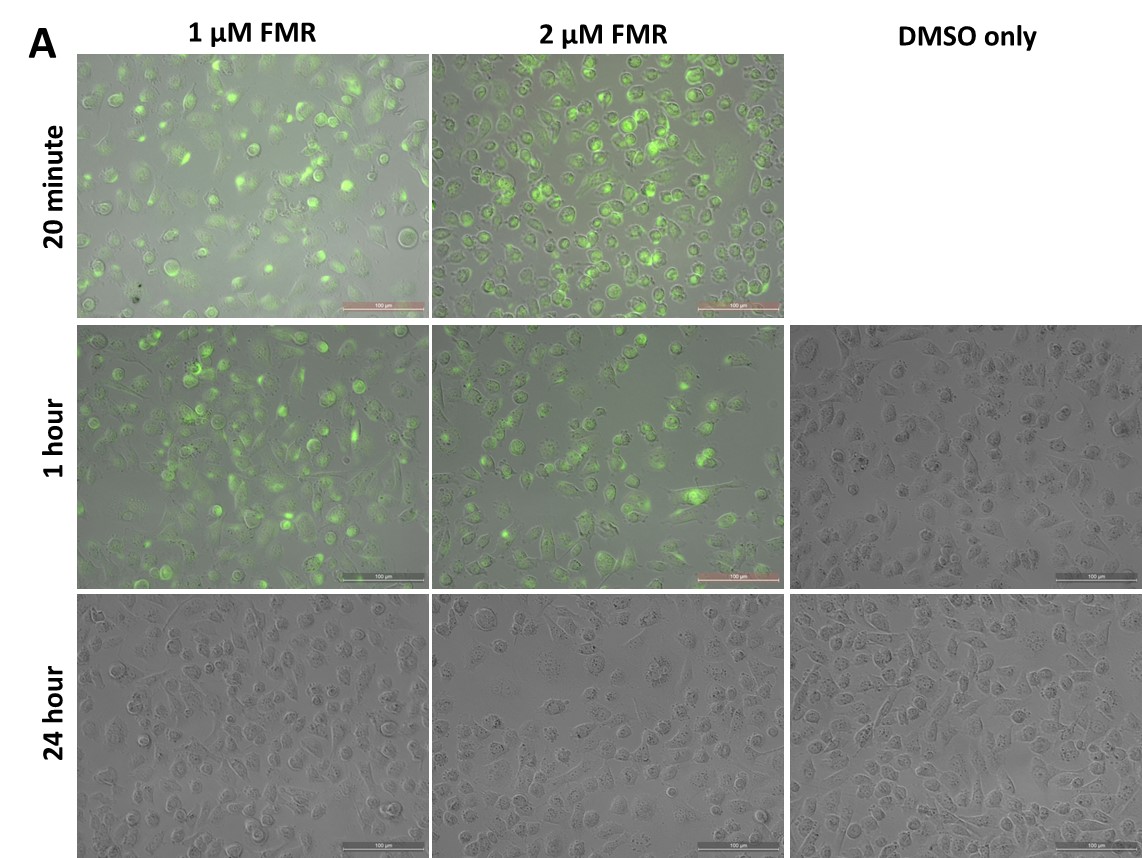


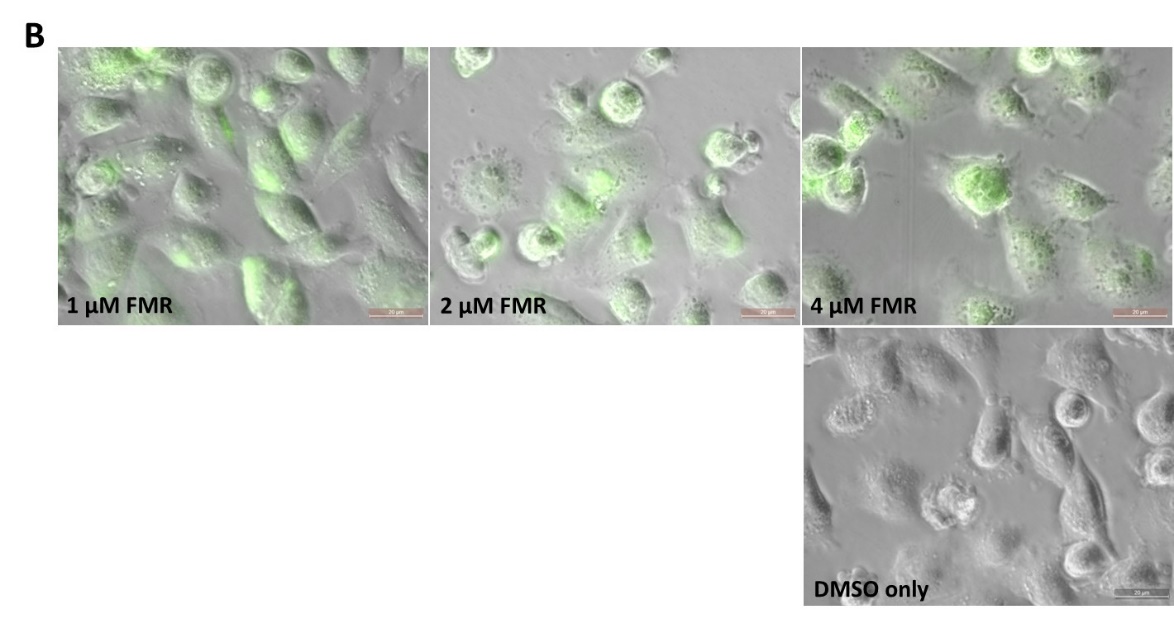


**SI8.** **Cell compatibility analysis: A)** MDA MB 231 cells with 1 µM, 2 µM and 4 µM FMR concentrations at 20-minutes, 1-hour and 24-hours; 20X, 100 µm bar **B)** MDA MB 231 cells with 1 µM, 2 µM and 4 µM FMR concentrations after 1-hour; 63X, 20 µm bar

**^1^H and ^13^C NMR spectra**


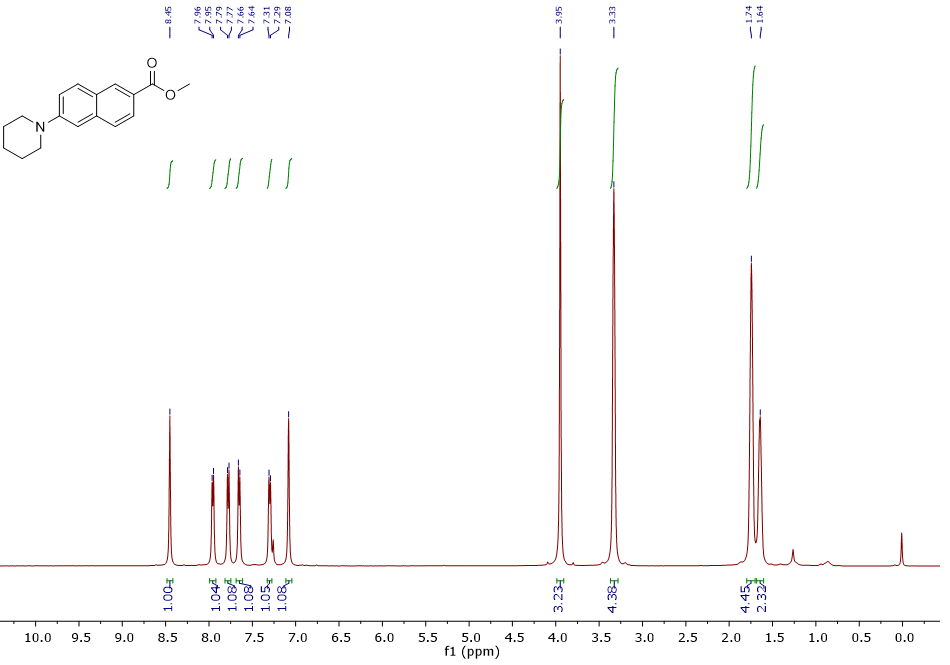


^1^H NMR (500 MHz, CDCl_3_) of compound **2**


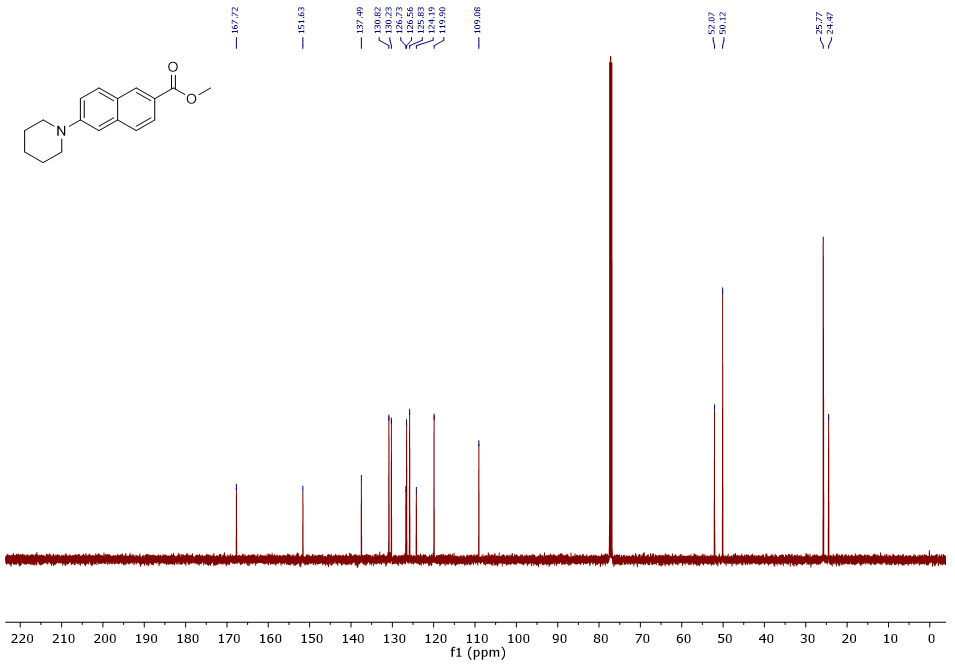


^13^C NMR (125 MHz, CDCl_3_) of compound **2**


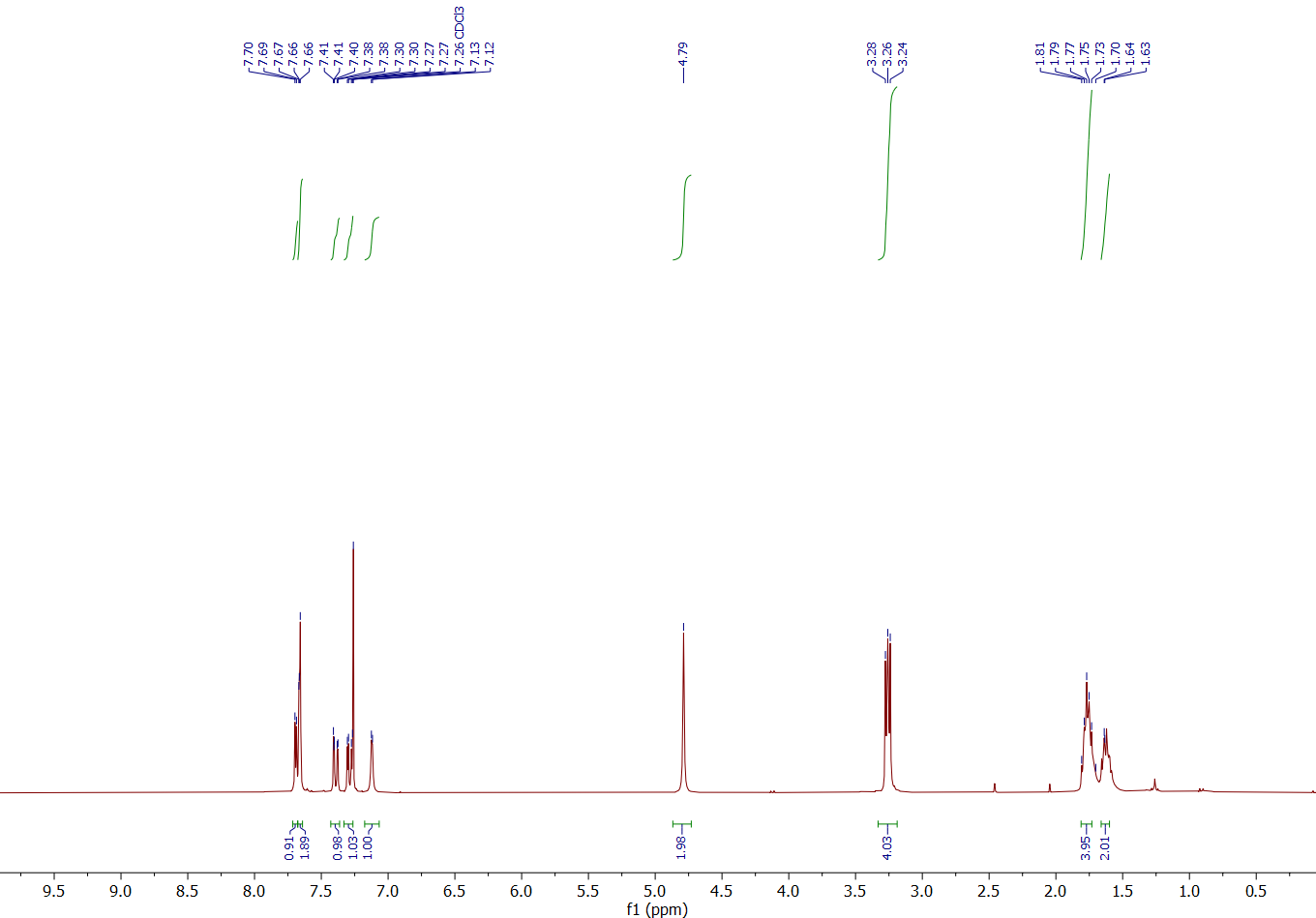

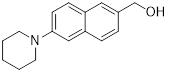


^1^H NMR (300 MHz, CDCl_3_) of compound **3**


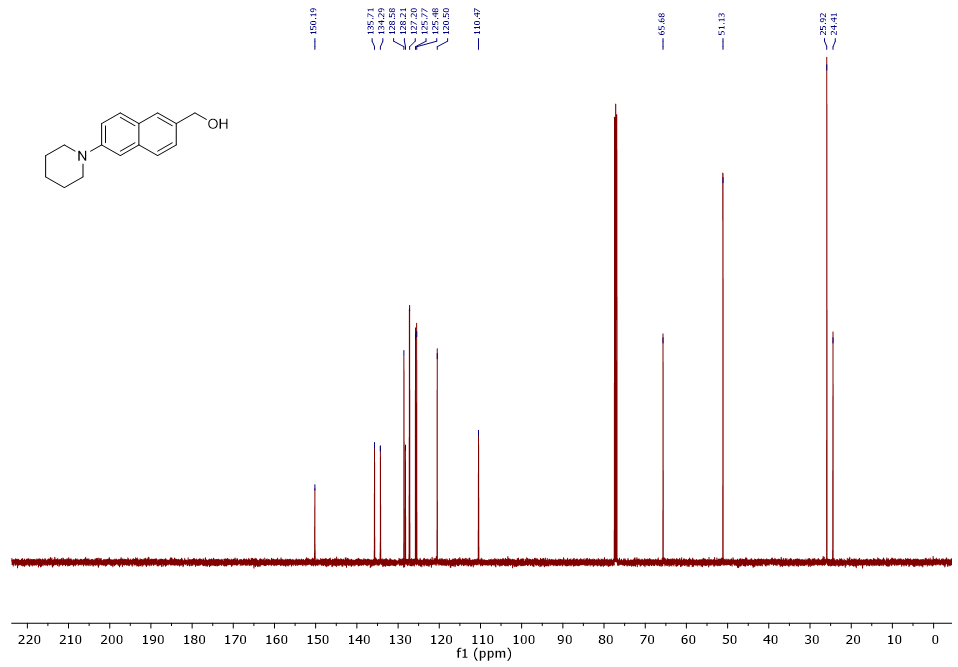


^13^C NMR (125 MHz, CDCl_3_) of compound **3**


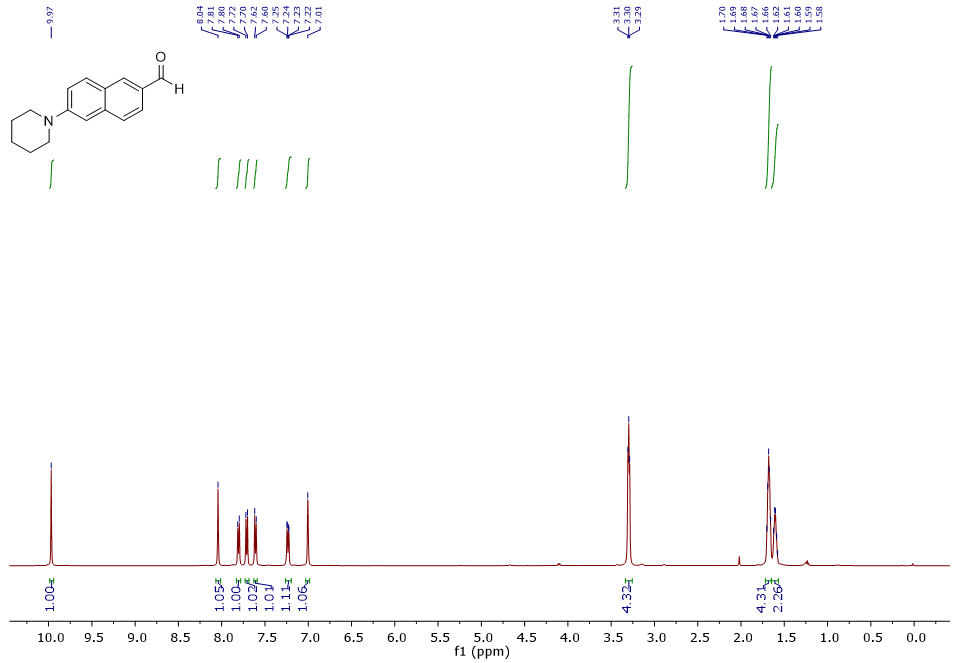


^1^H NMR (500 MHz, CDCl_3_) of compound **4**


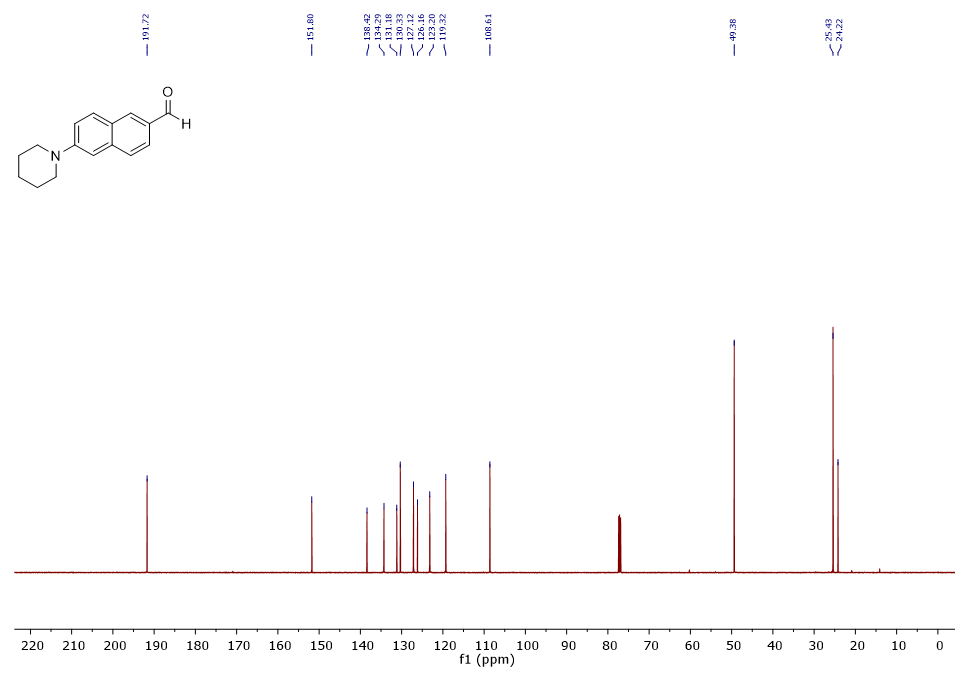


^13^C NMR (125 MHz, CDCl_3_) of compound **4**


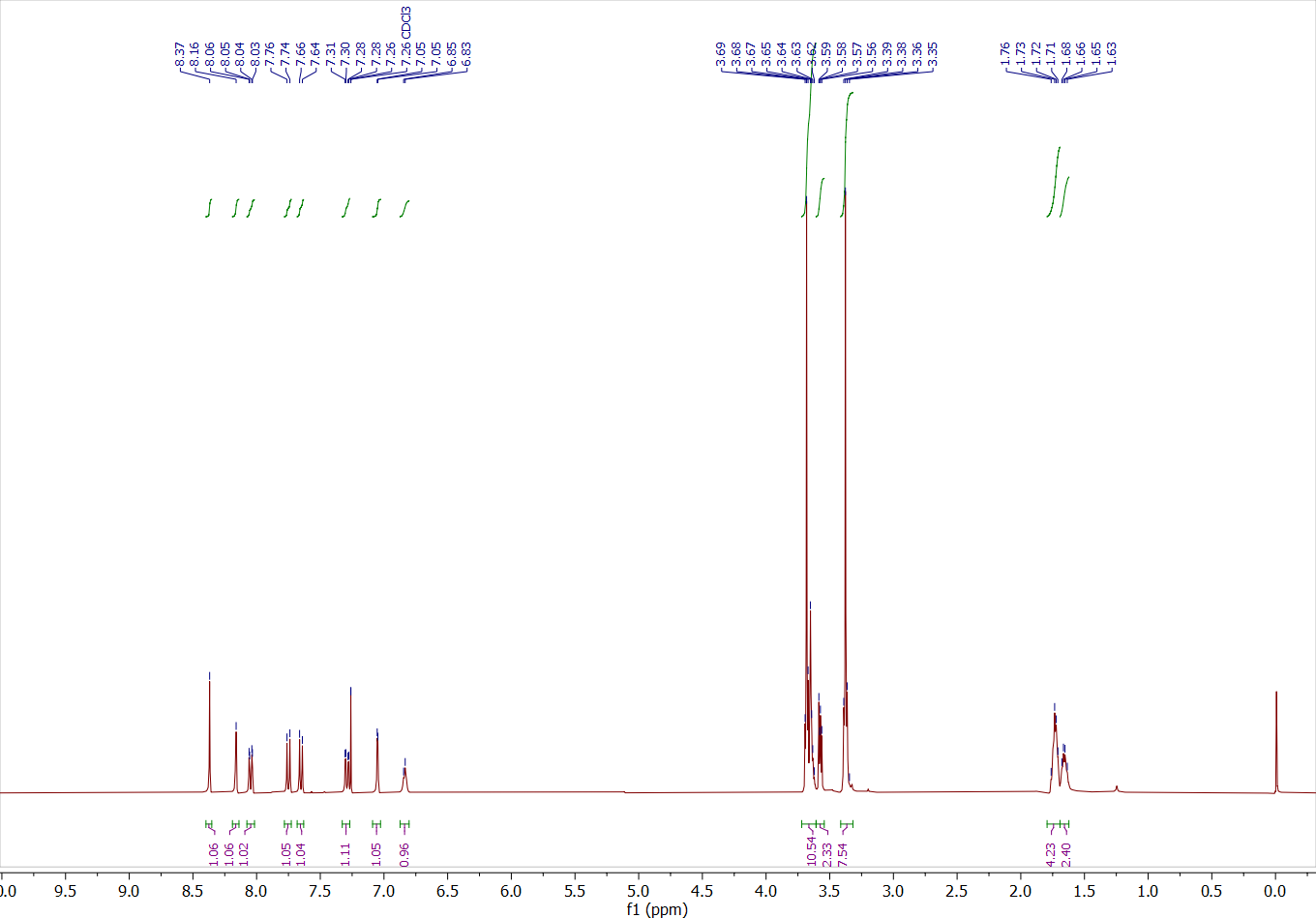


^1^H NMR (400 MHz, CDCl_3_) of **ARCAM-1**


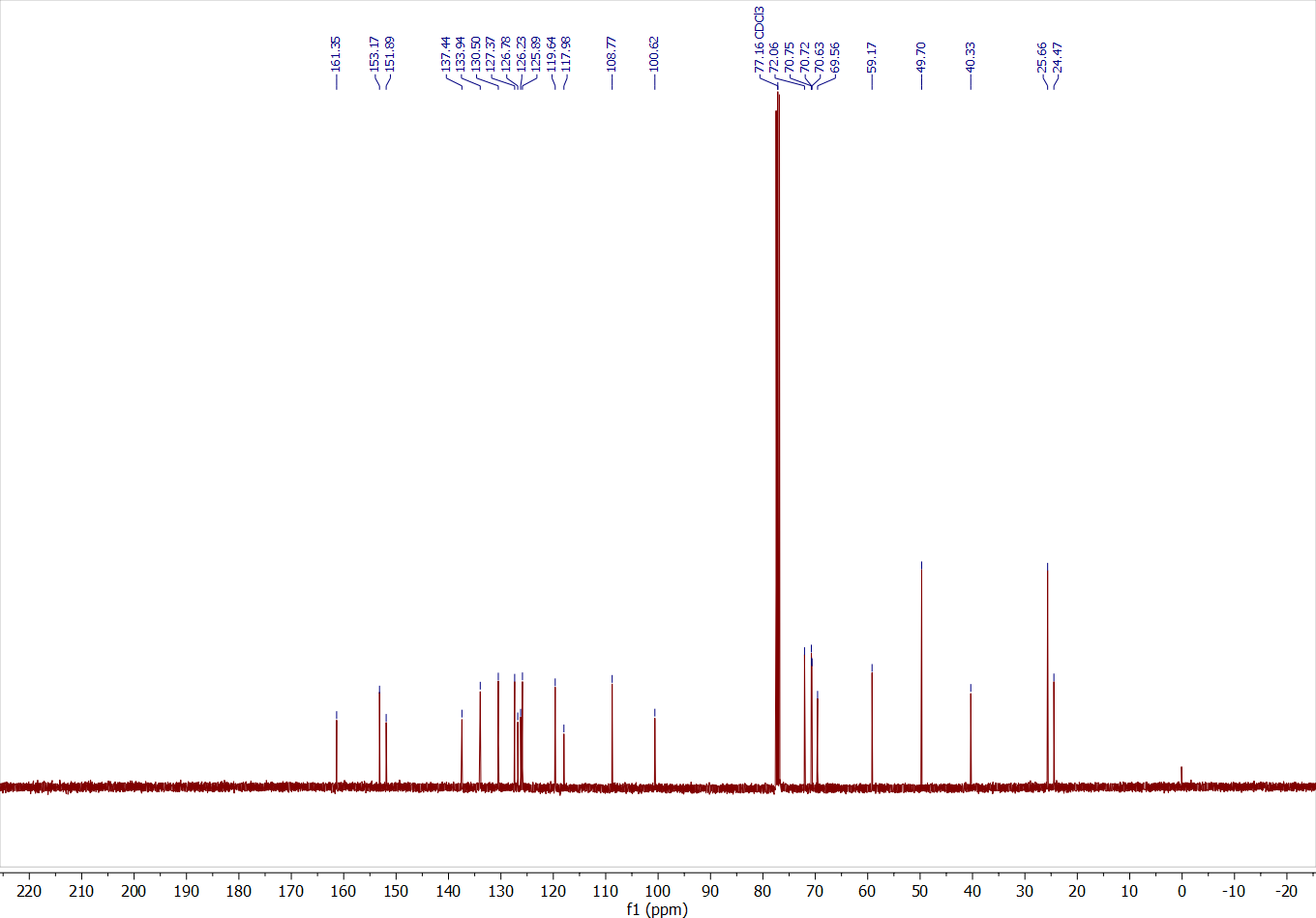


^13^C NMR (101 MHz, CDCl_3_) of **ARCAM-1**
